# Mechanical properties of metal–organic frameworks

**DOI:** 10.1039/c9sc04249k

**Published:** 2019-10-17

**Authors:** Louis R. Redfern, Omar K. Farha

**Affiliations:** a International Institute of Nanotechnology , Department of Chemistry , Northwestern University , 2145 Sheridan Road , Evanston , Illinois 60208 , USA . Email: o-farha@northwestern.edu

## Abstract

As the field of metal–organic frameworks (MOFs) continues to grow, the physical stability and mechanical properties of these porous materials has become a topic of great interest. While strategies for synthesizing MOFs with desirable chemical functionalities or pore sizes have been established over the past twenty years, design principles to modulate the response of MOFs to mechanical stress are still underdeveloped. The inherent porosity of these frameworks results in many interesting and sometimes unexpected phenomena upon exposure to elevated pressures and other physical stimuli. Beyond its fundamental importance, an understanding of mechanical properties (*e.g.* bulk modulus, shear modulus, Young's modulus, linear compressibility, and Poisson's ratio) plays an essential role in the post-synthetic processing of MOFs, which has implications in the successful transition of these materials from academic interest to industrial relevance. This perspective provides a concise overview of the efforts to understand the mechanical properties of MOFs through experimental and computational methods. Additionally, current limitations and possible future directions for the field are also discussed briefly.

## Abstract 2

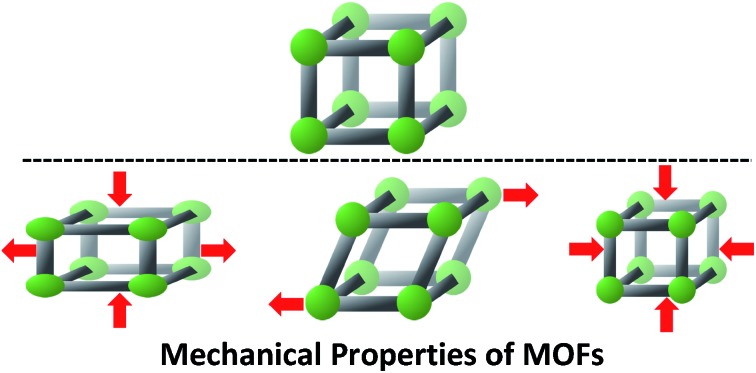
As the field of metal–organic frameworks (MOFs) continues to grow, the physical stability and mechanical properties of these porous materials has become a topic of great interest.

## 1. Introduction

Metal–organic frameworks (MOFs) are a class of porous, crystalline materials comprised of inorganic nodes joined by organic linkers. These components assemble into two- and three-dimensional networks with promising applications in a wide range of areas, including gas storage,^1^ catalysis,^2^ chemical separations,^3^ and drug delivery.^4^ Over the past twenty years, these highly functional materials have garnered much attention, with tremendous efforts put into the synthesis, characterization, and post-synthetic modification of these scaffolds; however, the study of the mechanical properties of MOFs (*e.g.* Young's modulus, shear modulus, bulk modulus, Poisson's ratio, and linear compressibility) is comparatively in its nascency. Beyond the fundamental importance of these properties, practical post-synthetic processing (*e.g.* extrusion and pellet formation) exposes the materials to significant mechanical stress.^5^ Understanding how MOFs respond to this stress is essential to their successful commercialization. With consistent synthetic protocols and well-defined crystal structures for thousands of MOF materials, researchers in this area are poised to make great strides in understanding the structure–property relationships that dictate the response of different MOFs to a variety of mechanical stresses.

The compression of porous materials has long been a fascinating topic of study, as the “empty” space in the structures yields surprising and unexpected behaviour at high pressures. Early efforts in this area investigated zeolites under high-pressure conditions.^6^–^10^ While zeolites and MOFs are notably distinct in many ways, prior analysis of the former helps to provide insight into the response of the latter to mechanical stress. Still, the inclusion of structural organic linkers in MOFs precludes the direct transfer of conclusions regarding zeolites to these hybrid materials. Likewise, although the fundamental advances in understanding the mechanical properties of MOFs is certain to influence the study of new porous materials (*e.g.* covalent organic frameworks), each class demands proper investigation.

The rich structural diversity of MOFs provides a near limitless number of interesting species to examine, but it comes with a cost in that the sheer number of distinct frameworks cannot be feasibly measured for every property. Given this limitation, it is important to consider structure–property relationships that can enable a more rapid and heuristic evaluation of materials design. Such relationships can only be drawn from careful, thorough studies that systematically vary a single structural characteristic of a MOF. Unfortunately, experimental data are often complicated by subtle differences between MOFs resulting from batch-to-batch variance (*e.g.* defect density, porosity, and guest loading). Moreover, many experiments rely on advanced techniques such as diamond anvil cell (DAC) sample environments and synchrotron radiation which puts significant constraints on the availability of such experimental data. Fortunately, computational simulation is a powerful tool for studying the mechanical properties of MOFs and has been used extensively in stand-alone and joint investigations.^11^,^12^ These two complementary approaches are essential for the successful analysis of the mechanical properties of MOFs.

The examples highlighted in this review are selected to summarize many of the major advances and efforts toward understanding the mechanical properties of MOFs over the past decade. The linker, node, and structure of each MOF discussed herein is displayed in Table 1. Several reviews on this topic have been written that delve into the fine details of individual investigations;^13^–^16^ however, this perspective is intended to provide a broad context of the field while discussing in depth several reports that stand out in the field. Furthermore, this work is not meant to be a tutorial for conducting high-pressure experiments, as this is covered extensively elsewhere.^17^ The studies discussed herein are organized by into broad classes of MOFs, emphasizing the often varied results obtained from subtle differences in experimental design, even when examining closely related or identical materials.

**Table 1:** MOF components and structures discussed in this review

MOF	Organic linker	Inorganic node	Structure	Ref.
ZIF-3	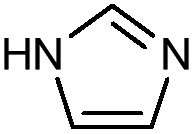	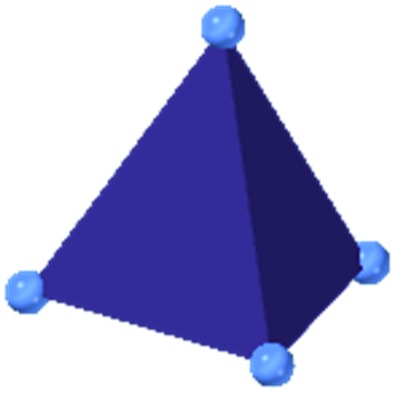	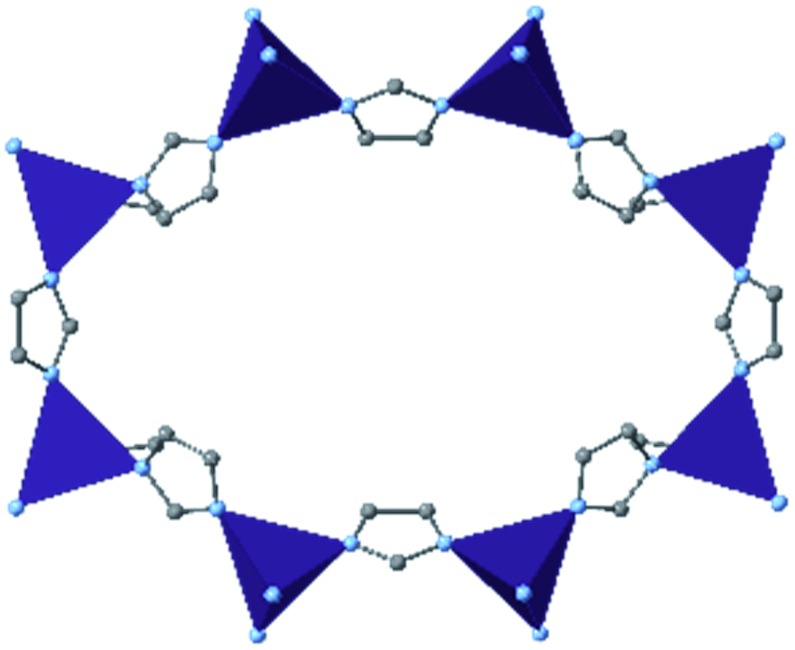	25
ZIF-4	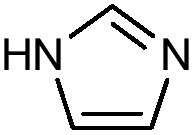	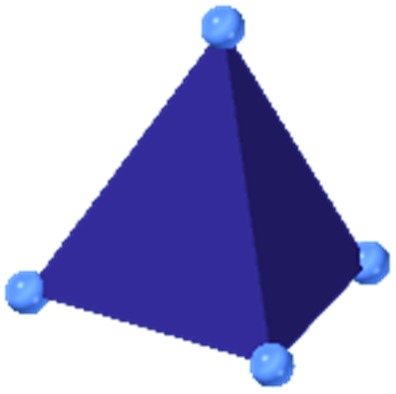	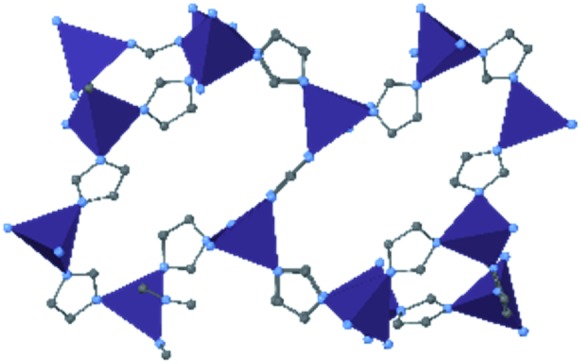	22–24 and 27–29
ZIF-8	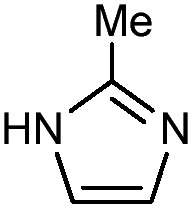	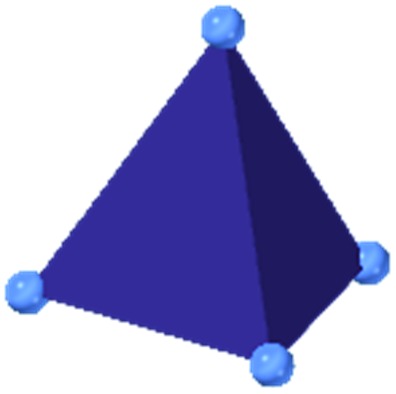	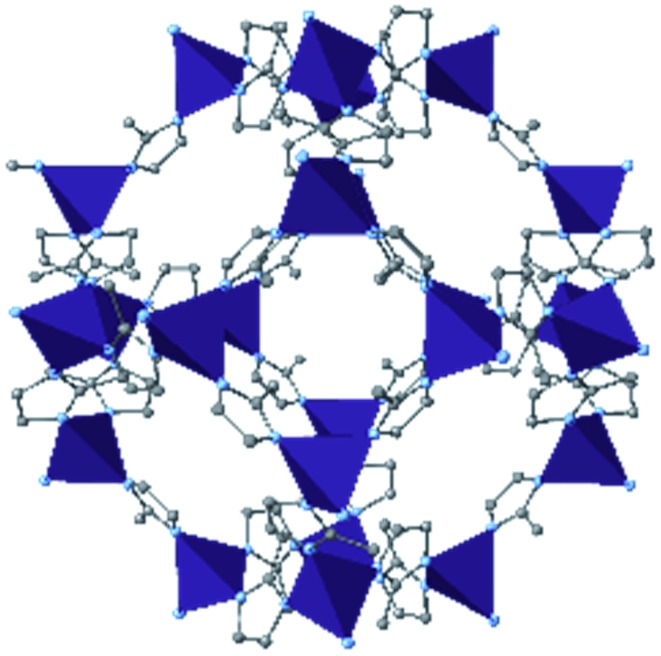	20–22, 24, 26, 27 and 35
ZIF-62	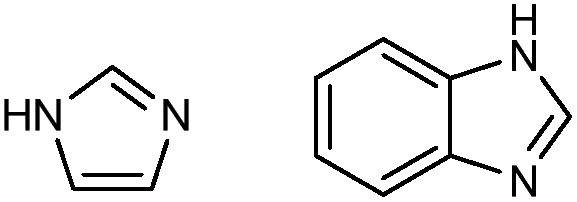	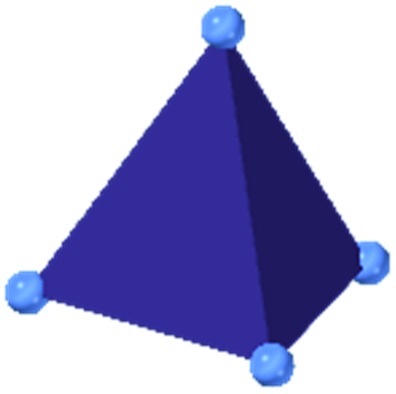	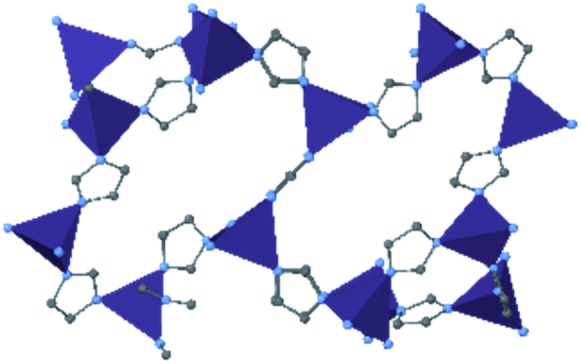	29
HKUST-1 (Cu-BTC)	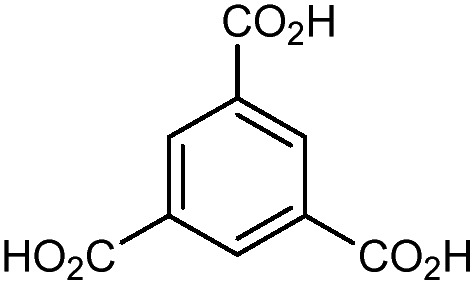	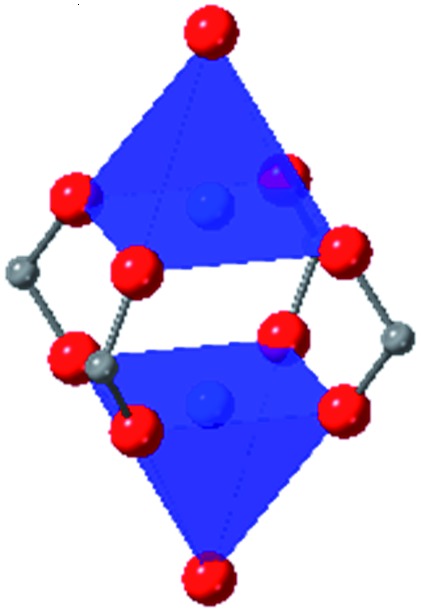	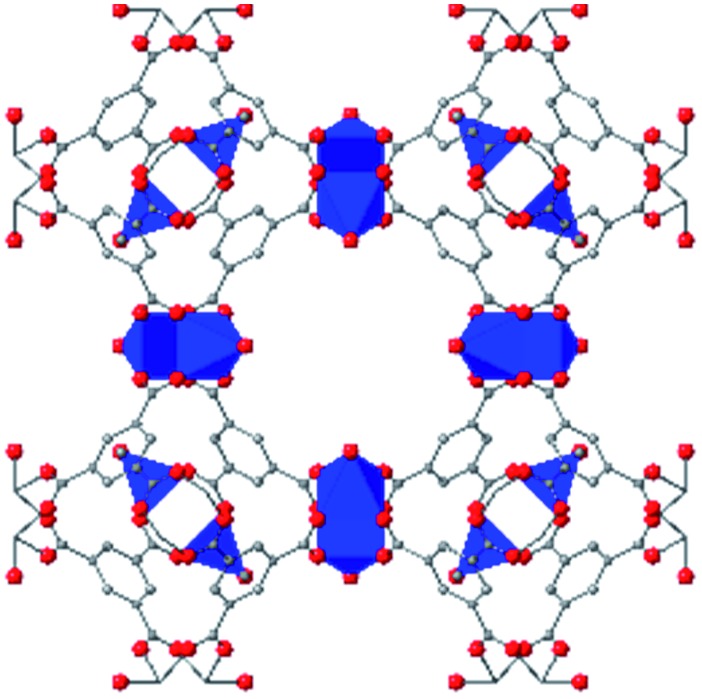	37–42
MOF-5 (IRMOF-1)	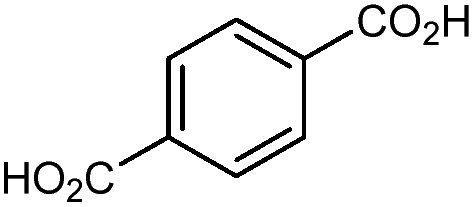	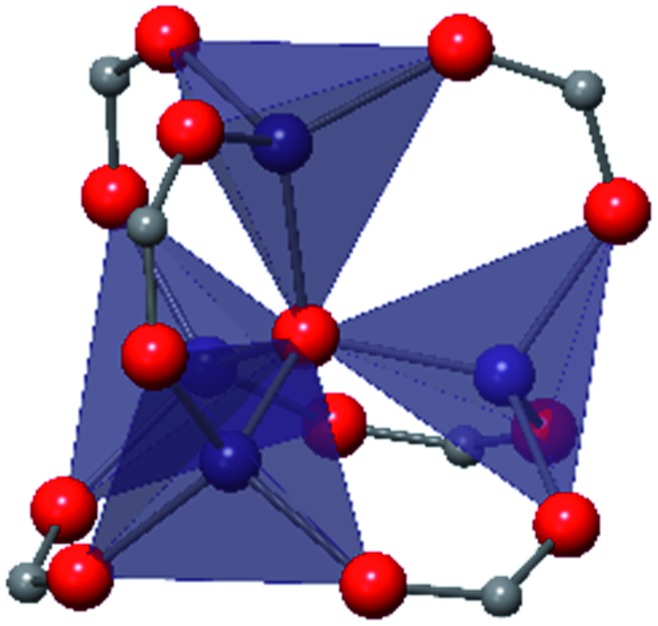	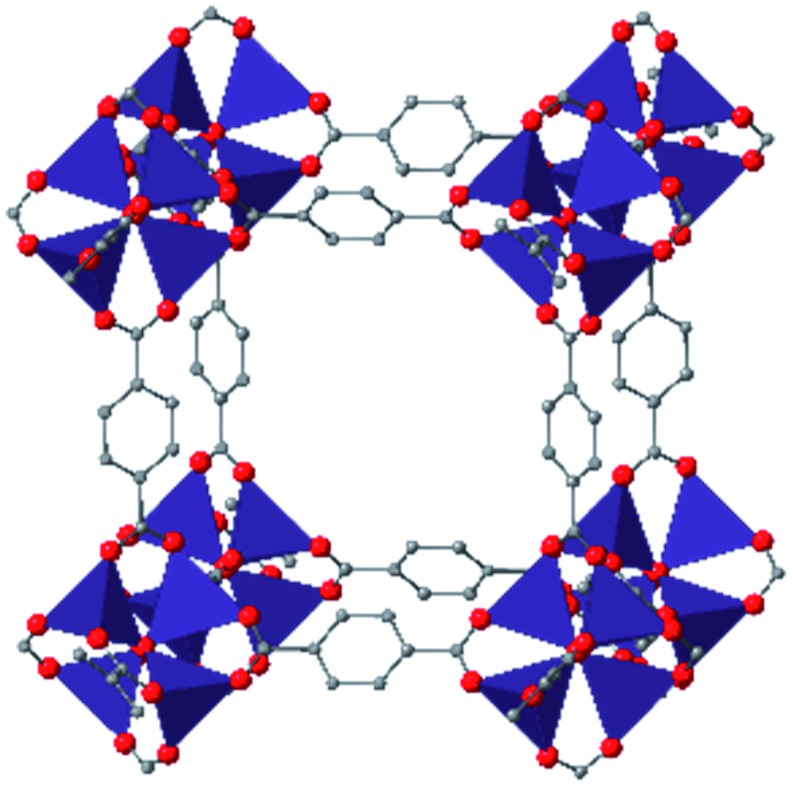	43–47
MIL-53(M)	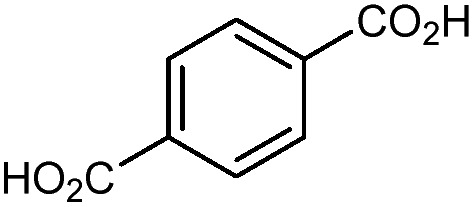	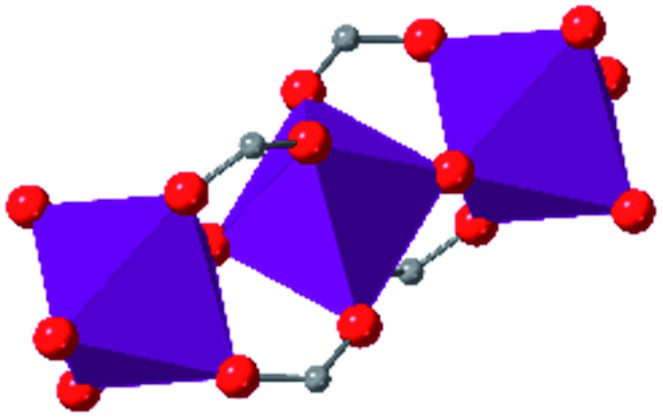	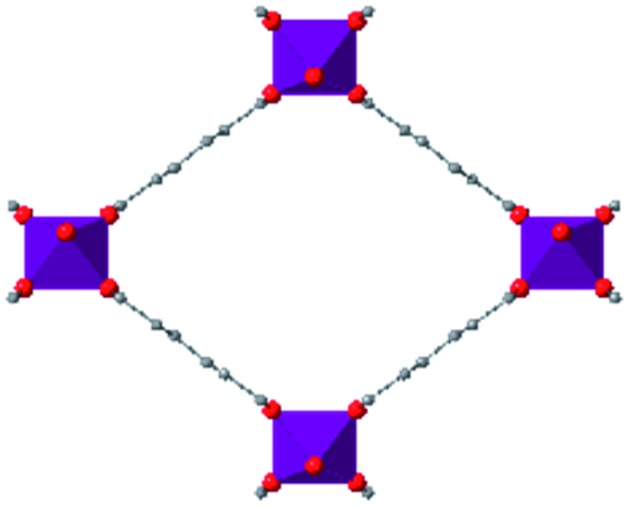	48–54
UiO-66	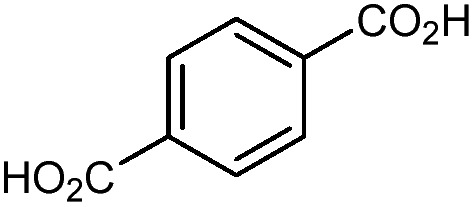	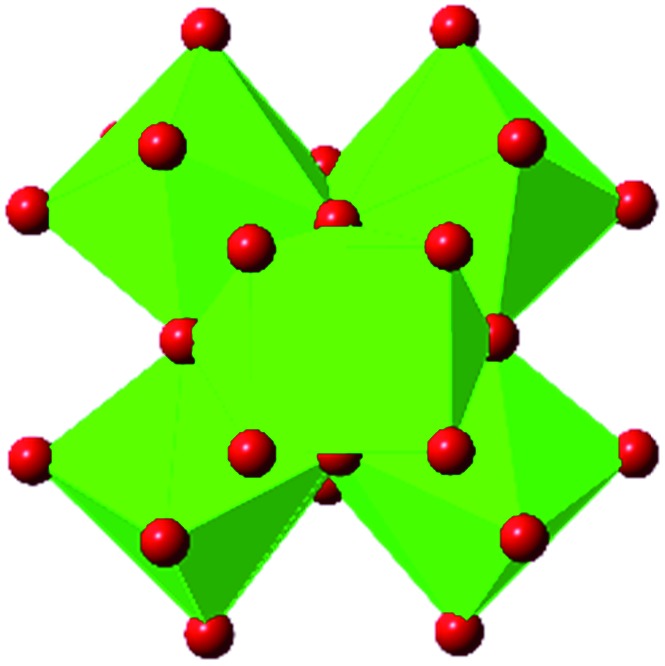	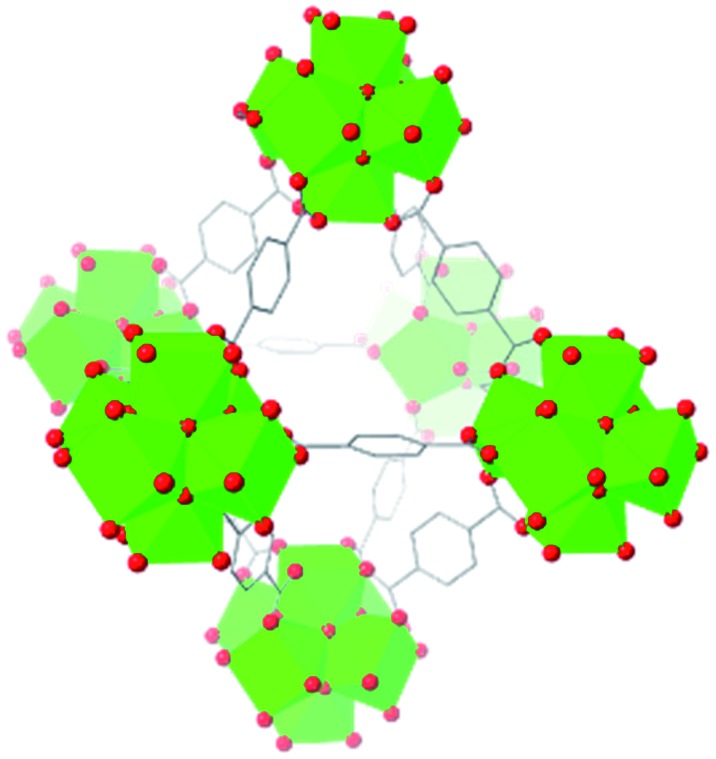	16, 40, 57–62 and 67
UiO-67	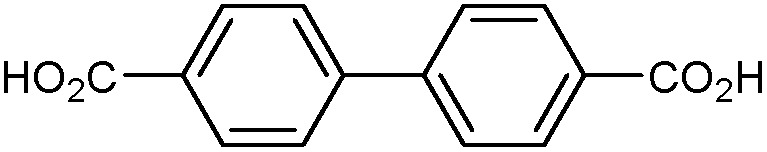	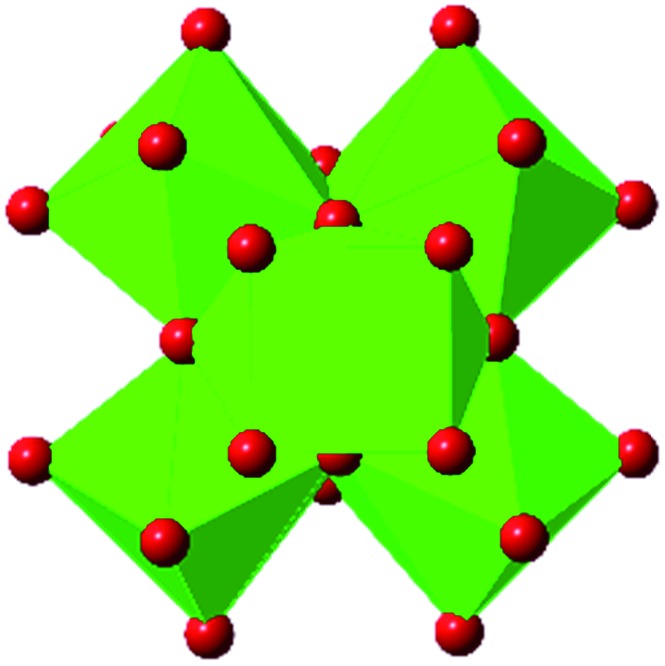	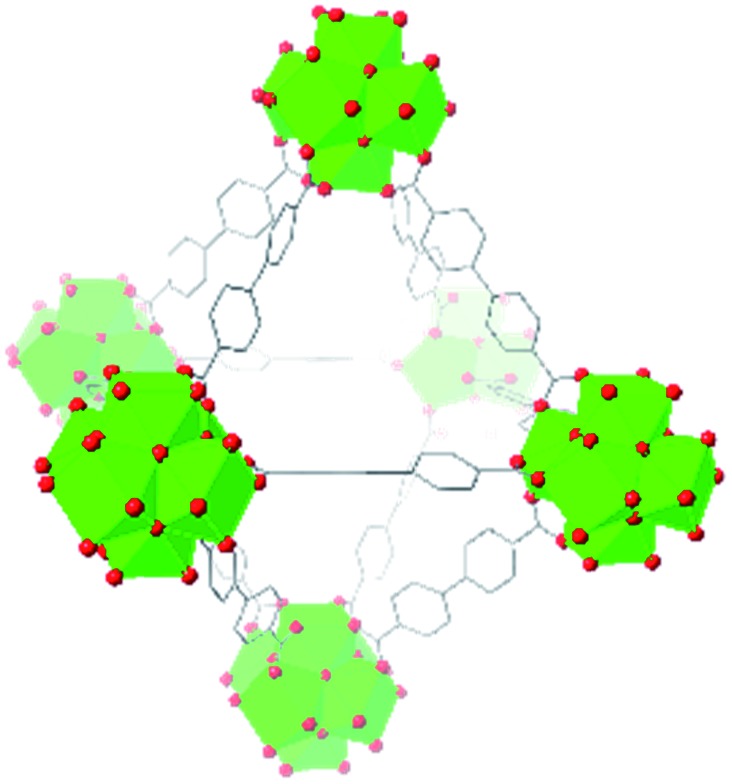	63 and 67
UiO-abdc	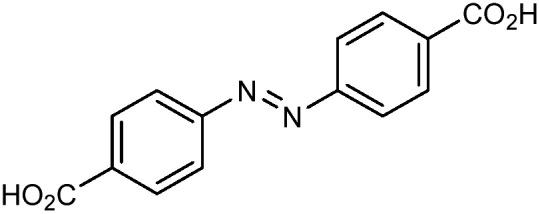	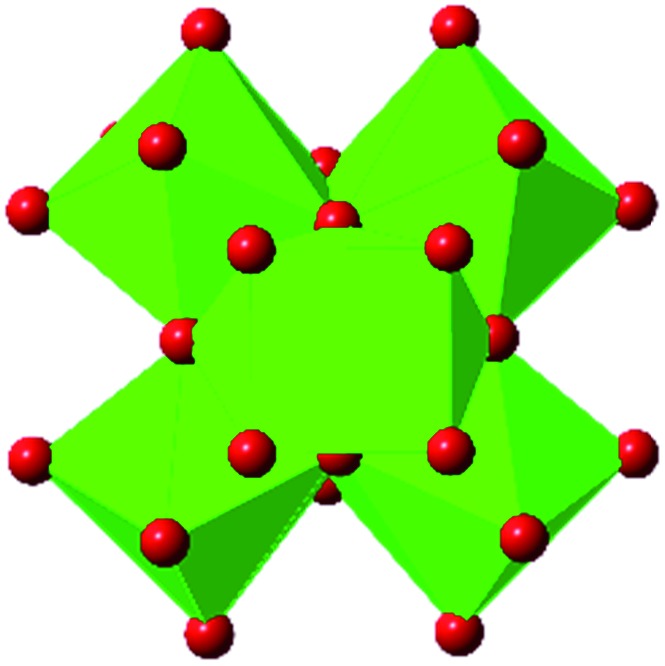	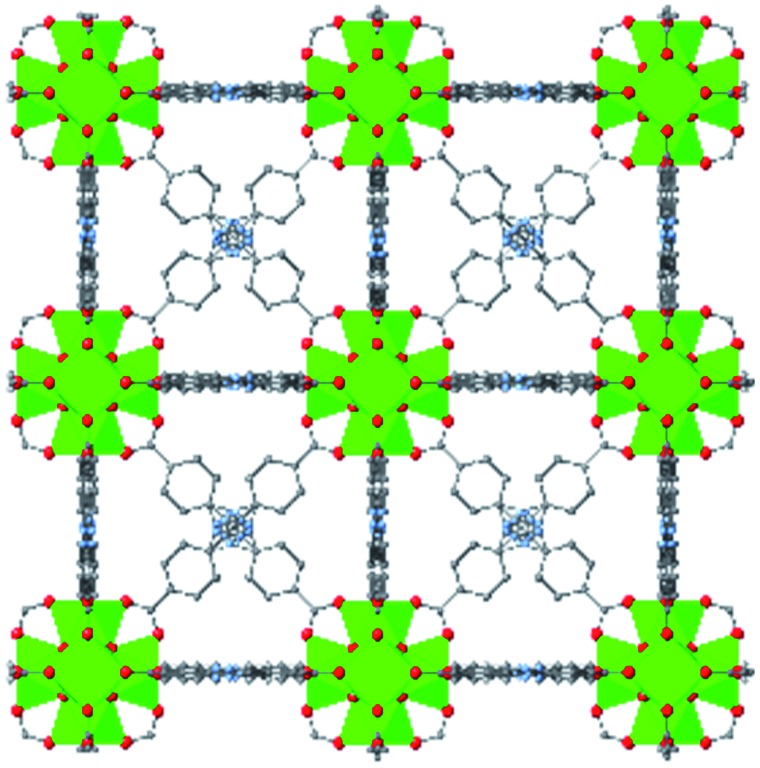	63
Zr_6_O_4_(OH)_4_(edb)_6_ (**1**)	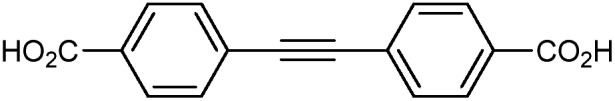	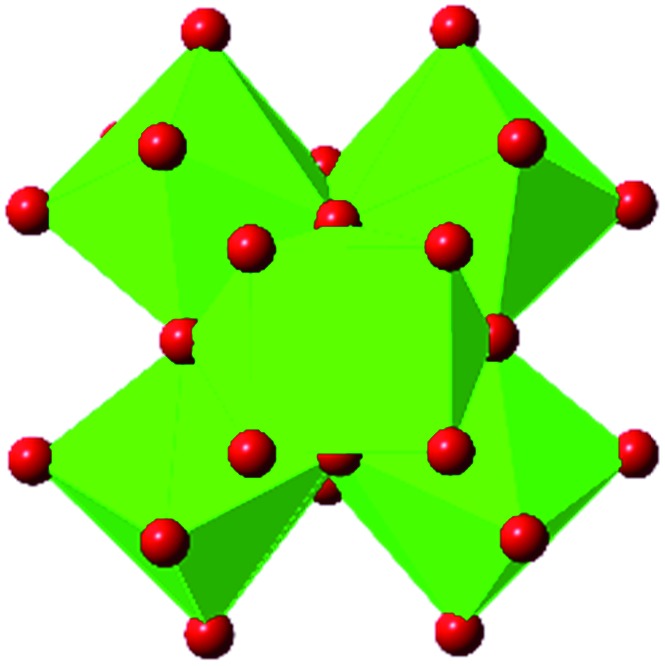	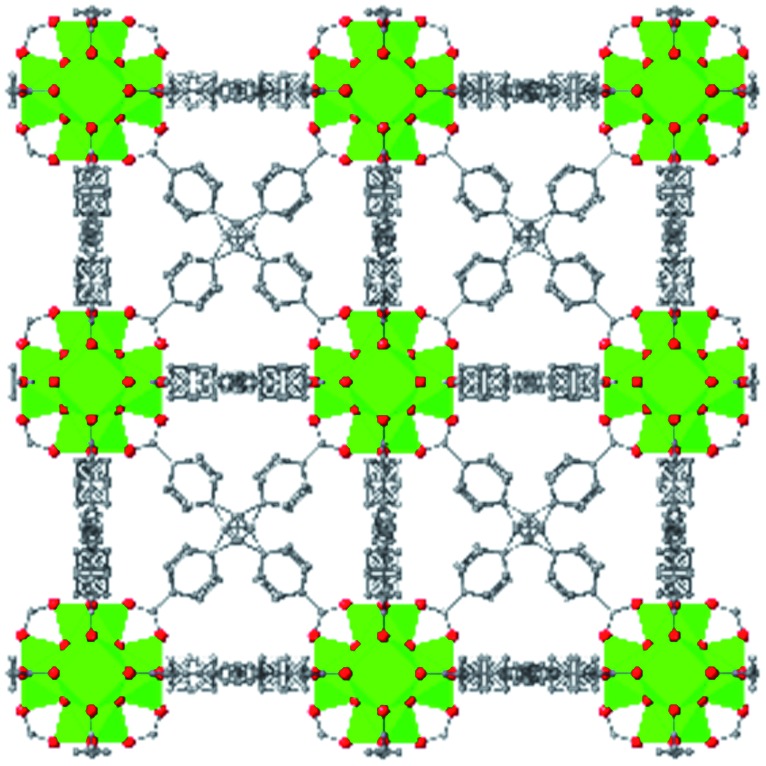	64
Zr_6_O_4_(OH)_4_(sdc)_6_ (**2**)	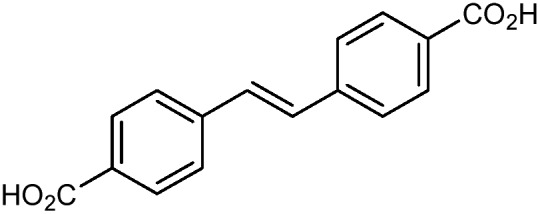	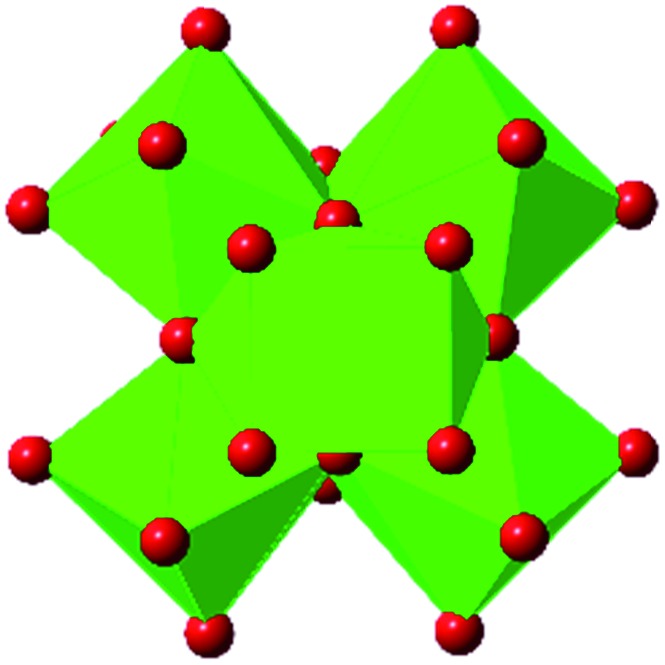	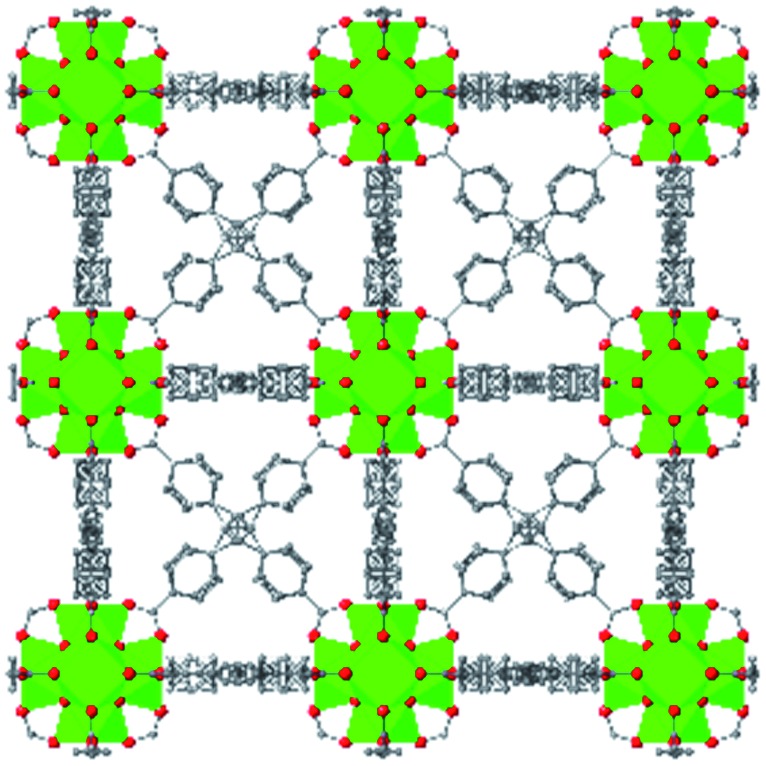	64
MIL-140	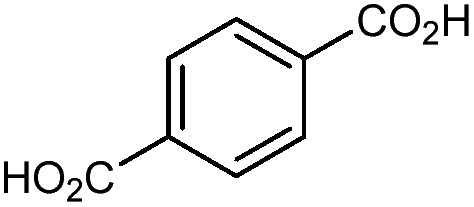	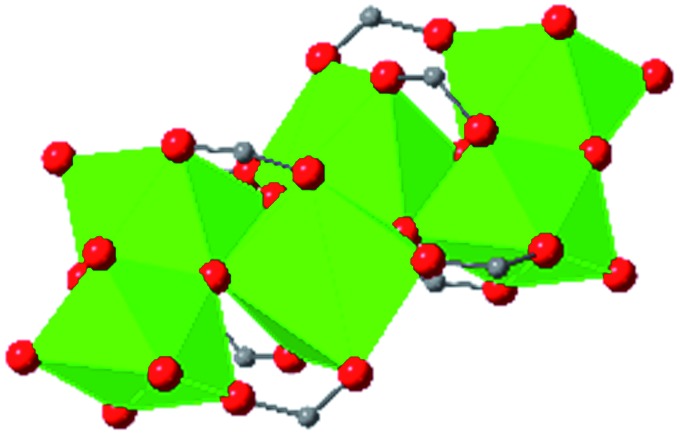	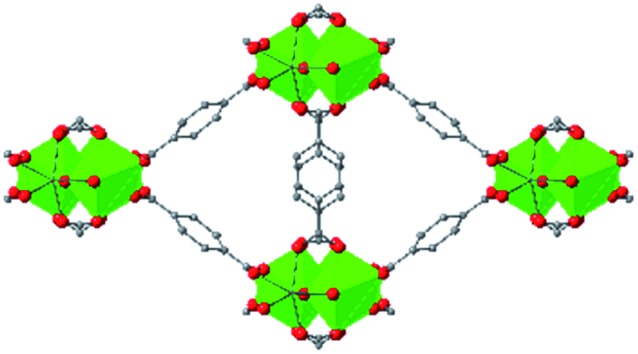	65 and 66
NU-901	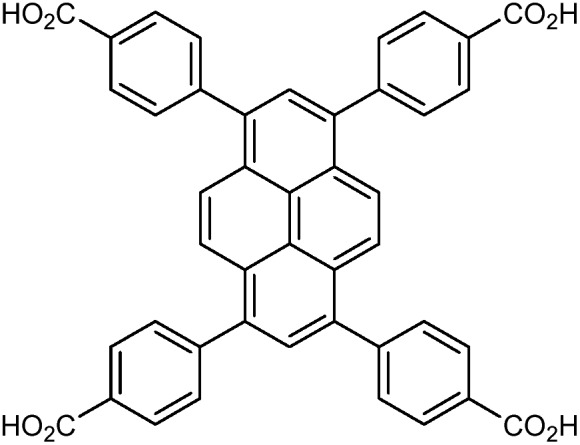	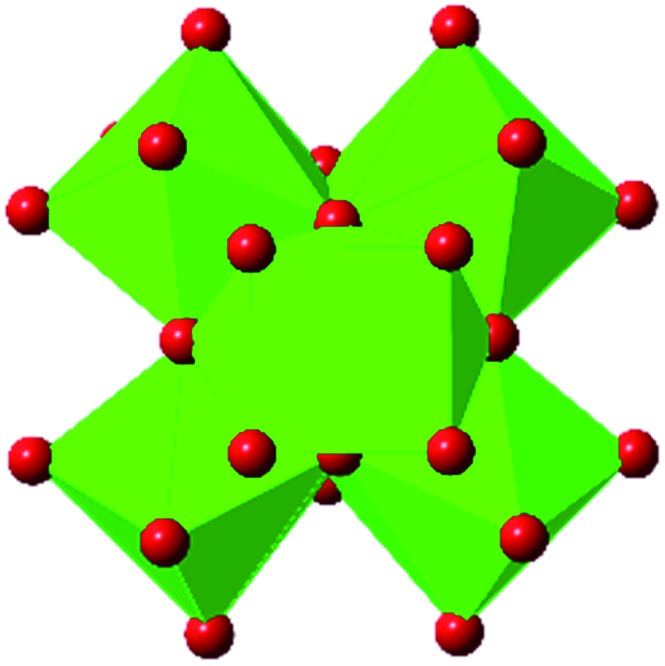	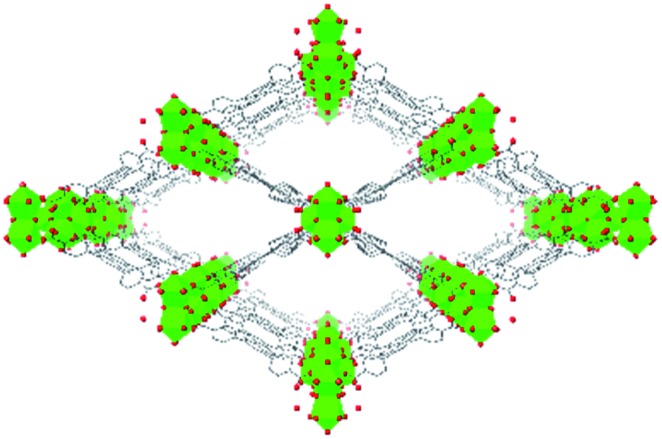	67
Co_2_(4,4′-bpy)_3_(NO_3_)_4_ (**3**)	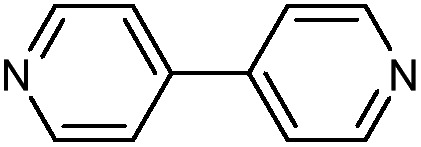	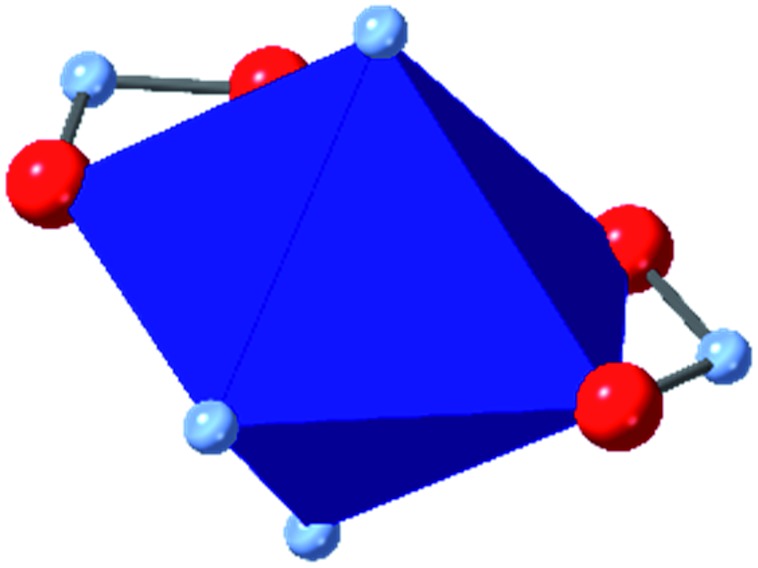	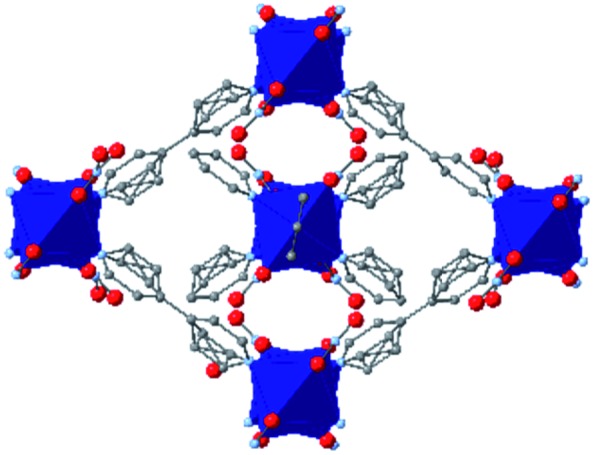	70
Zn_2_(L)_2_(dabco) (**4**)	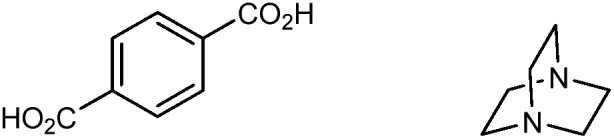	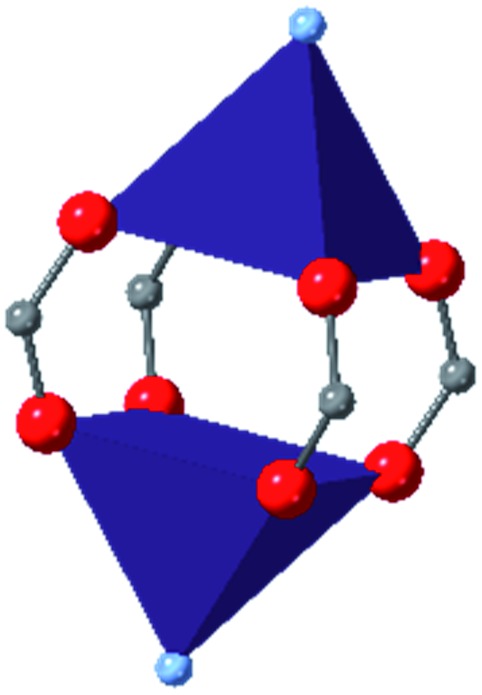	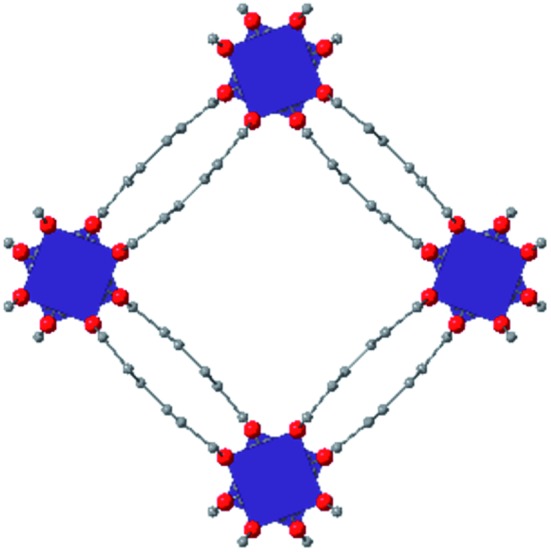	71
MOF-74	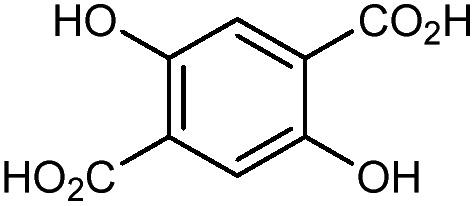	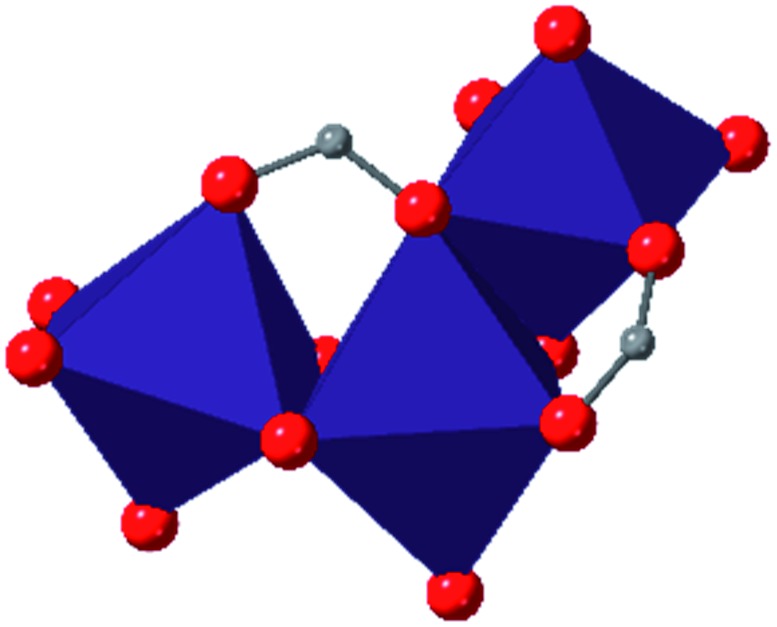	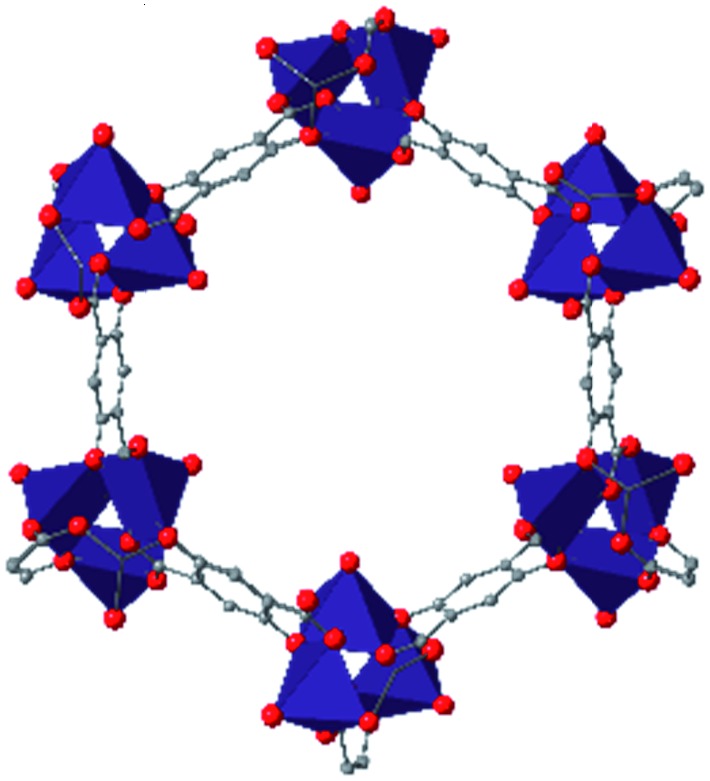	72 and 73
Cu_24_ isophthalate (L1/L2) (**5**)	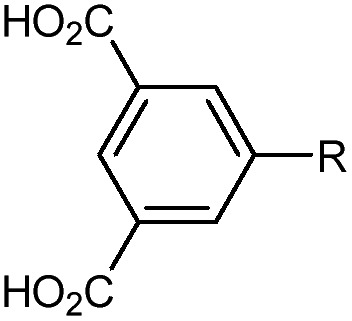	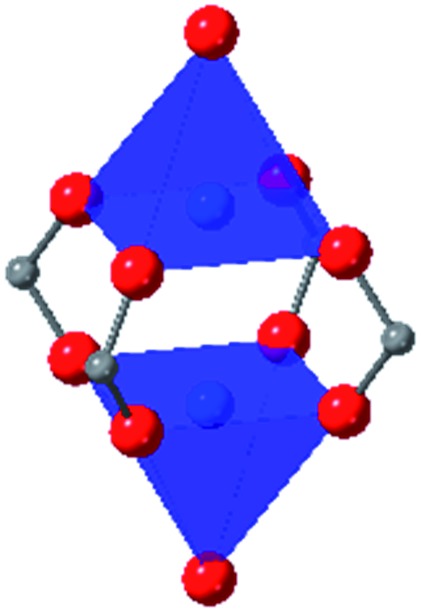	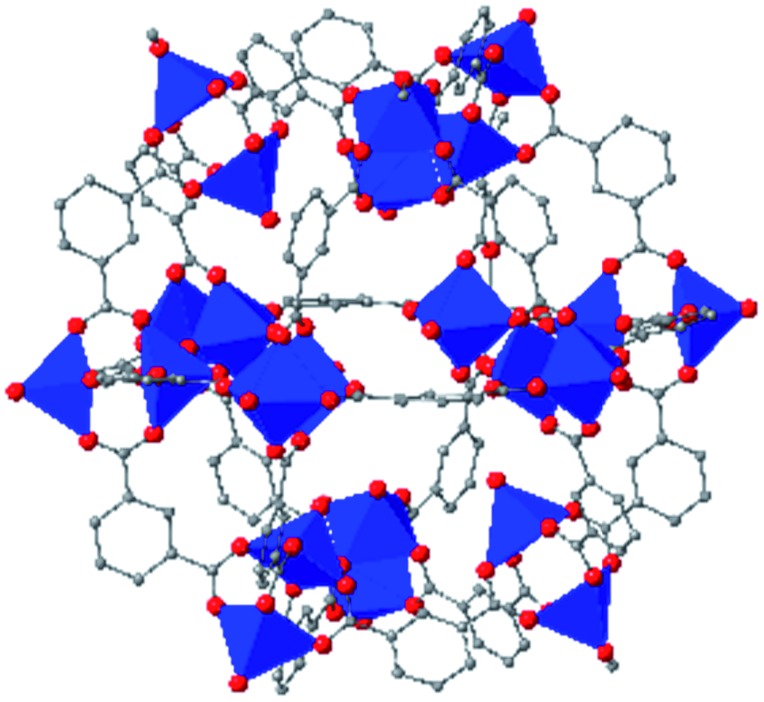	74
DUT-60	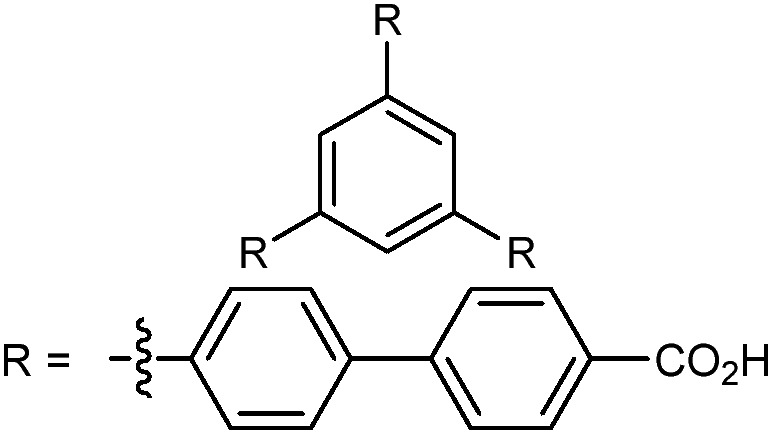	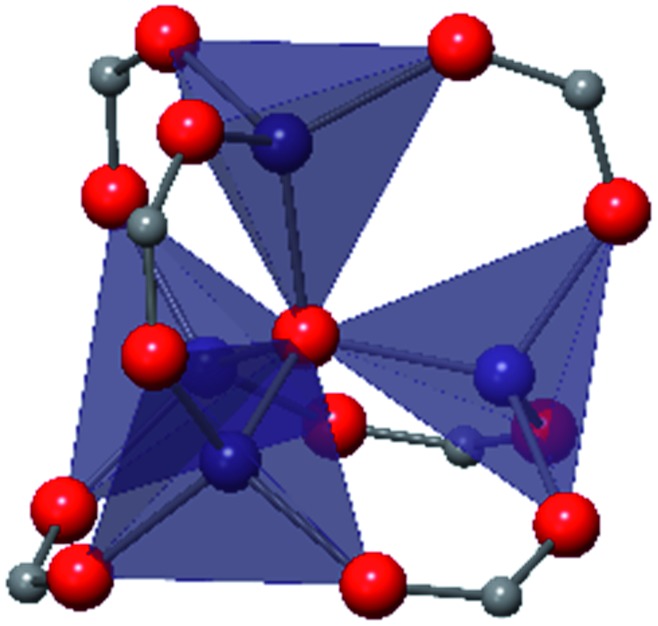	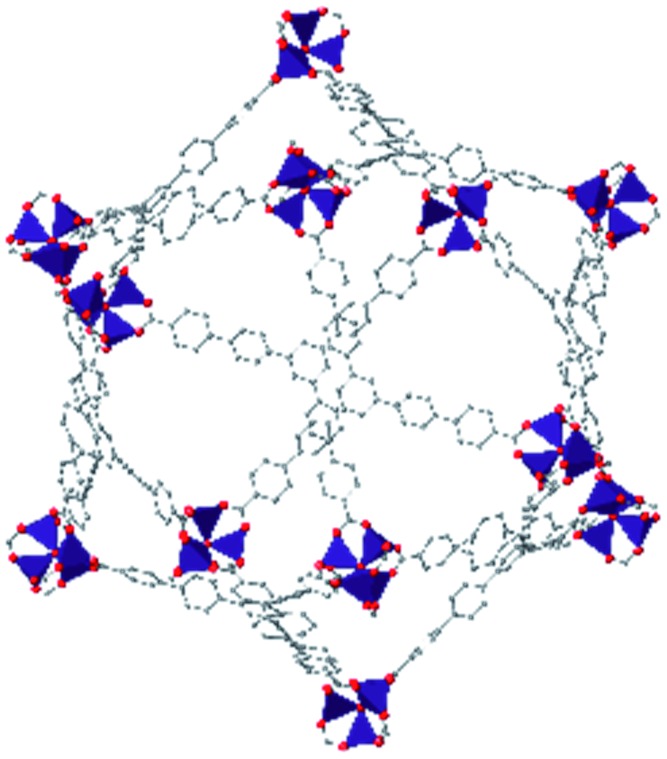	75
Sc_2_(BDC)_3_	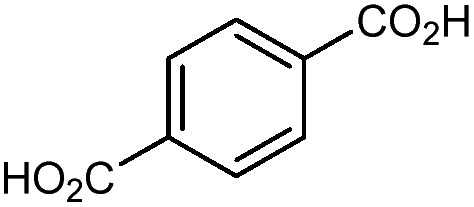	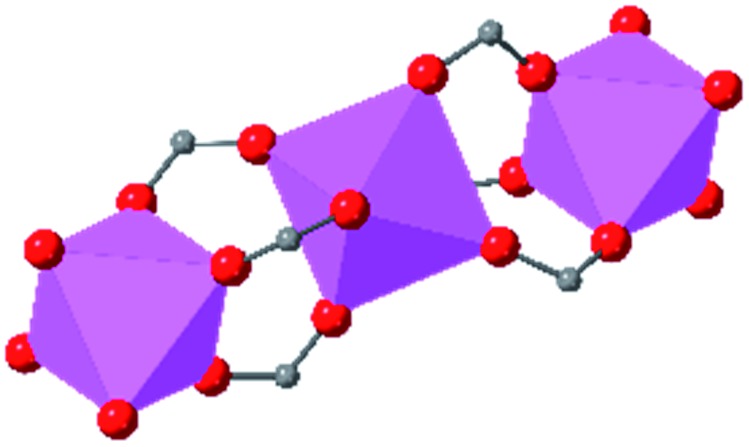	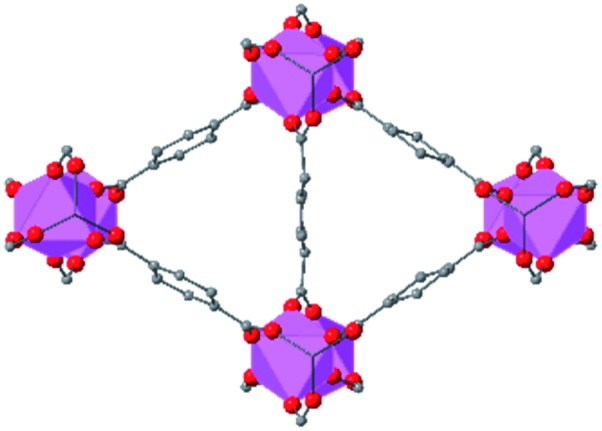	77
MOF-520	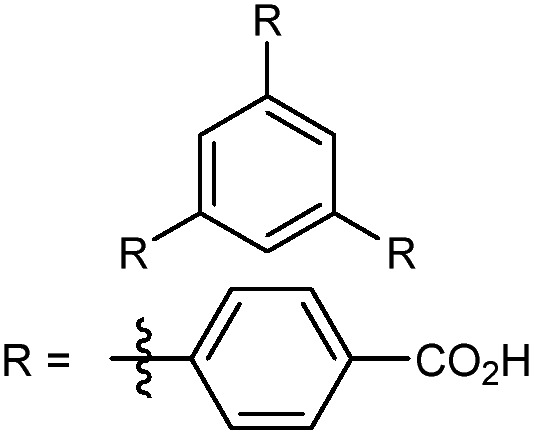	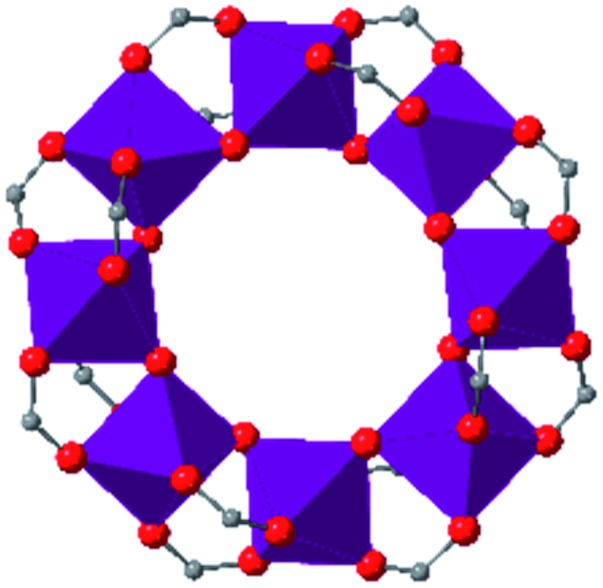	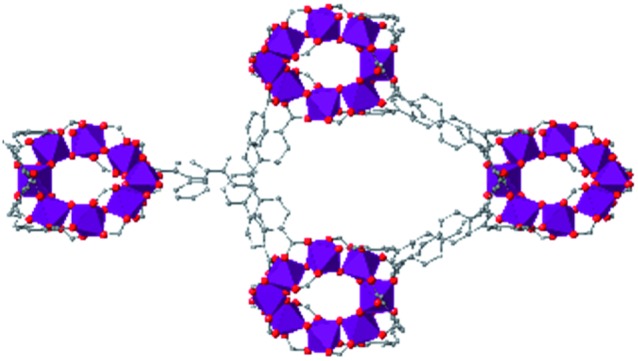	80

## 2. Zeolitic imidazolate frameworks (ZIFs)

Among the most famous classes of MOFs, zeolitic imidazolate frameworks (ZIFs), are nanoporous materials comprised of tetrahedrally-coordinated single-ion nodes and imidazole-derived organic linkers. The metal–imidazole–metal linkage of these frameworks resembles the Si–O–Si angles present in zeolites, giving rise to their name. Due to their facile synthesis,^18^ stability under ambient conditions,^19^ and commercial availability of linkers and pre-synthesized materials, ZIFs are one of the most well-studied classes of MOFs regarding mechanical properties. We provide here a brief overview of many studies conducted in the past decade, followed by a more in-depth analysis of several milestone investigations that continue to influence our understanding of the mechanical properties of these materials.

Since the first reports of DAC diffraction experiments of ZIF-8 [Zn(2-methylimidazolate)_2_] in 2009,^20^,^21^ many groups have examined this diverse class of MOFs to understand their behaviour at high pressure and temperature. Cheetham and coworkers found that across seven distinct materials, the elastic modulus and hardness tend to decrease as solvent accessible volume increases,^22^ and reported the high-pressure phase of ZIF-4 [Zn(imidazolate)_2_] following a single-crystal X-ray diffraction (SCXRD) study.^23^ In addition to experimental efforts, computational analysis has provided invaluable insight into the mechanism and implications of high pressure on MOFs. Coudert and coworkers probed the compression of ZIF-4 and ZIF-8, finding that amorphization of these MOFs could be explained by invoking a “shear-mode softening” mechanism.^24^ Ryder and Tan sought to isolate the role of topology in the mechanical properties of a series of MOFs with identical chemical components. Their findings demonstrate that the spatial orientation of nodes and linkers can have a significant impact on the stability of MOFs, as evidenced by the exceptionally low Young's and shear moduli of ZIF-3 [Zn(imidazolate)_2_].^25^ Modifications to the electronic properties^26^ and steric bulk^27^ of the organic linkers have been shown to enhance or diminish the various mechanical properties of the porous frameworks. Recent research interest in the concept of liquid- and glass-phase MOFs has brought about redoubled efforts to map out the high-pressure–high-temperature phase space of ZIF-4 (ref. 28) and ZIF-62 [Zn(benzimidazolate)_0.25_(imidazolate)_1.75_].^29^

**Fig. 1:**
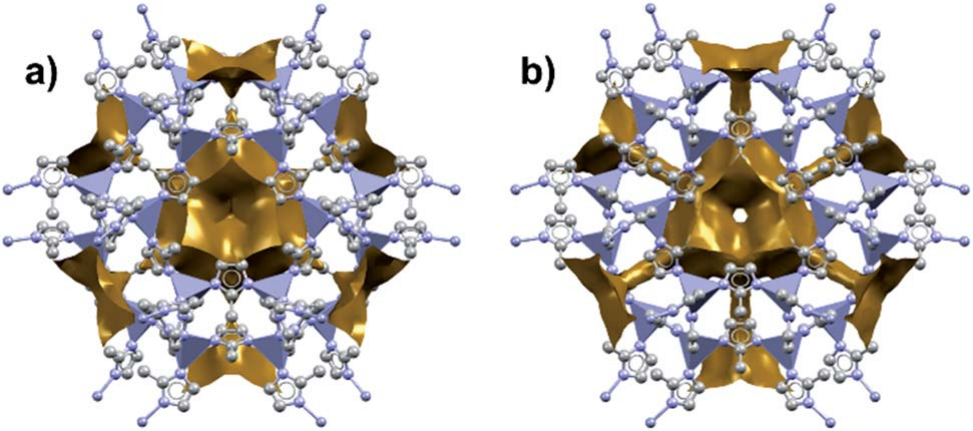
Accessible pore volume of ZIF-8 at (a) 0 GPa and (b) 1.47 GPa. The twist of the imidazolate ligands opens new channels of porosity within the structure. Adapted with permission from ref. 20. Copyright 2009, Wiley-VCH.

Among the first experimental investigations of ZIFs under high pressures, Moggach, Bennett, and Cheetham conducted a SCXRD study on ZIF-8 in a DAC.^20^ High-pressure diffraction data were collected with a mixture of methanol and ethanol as a pressure transmitting fluid. Upon raising the pressure to 0.18 GPa, the crystal structure swelled unexpectedly rather than compressing. This counterintuitive phenomenon of unit cell expansion under pressure is now found throughout the literature and is attributed to “hyperfilling” of the MOF pores with the hydrostatic fluid, enlarging the framework at moderate pressures. At 1.47 GPa, the ZIF-8 sample underwent a single-crystal to single-crystal phase transition in which the imidazolate ligands twist and increase the accessible pore volume (Fig. 1). Shortly after this report, Chapman, Halder, and Chupas published their own study utilizing powder X-ray diffraction (PXRD) of ZIF-8 in a DAC using Fluorinert™ FC-75 as a nonpenetrating pressure transmitting fluid. Under these conditions, the bulk modulus (*K*_0_) was estimated to be 6.5(4) GPa, with clear amorphization of the material at pressures beyond 0.34 GPa.^21^ In addition to diffraction studies, Chapman *et al.* monitored the porosity of pressure-treated ZIF-8 using nitrogen adsorption, revealing a precipitous drop in surface area and pore volume following exposure to pressure. The stark contrast between the behaviour of ZIF-8 in the studies of Chupas and Cheetham is due to the choice of hydrostatic fluid—FC-75 is too large to fill the pores of ZIF-8 and support the framework, leading to rapid amorphization.

**Fig. 2:**
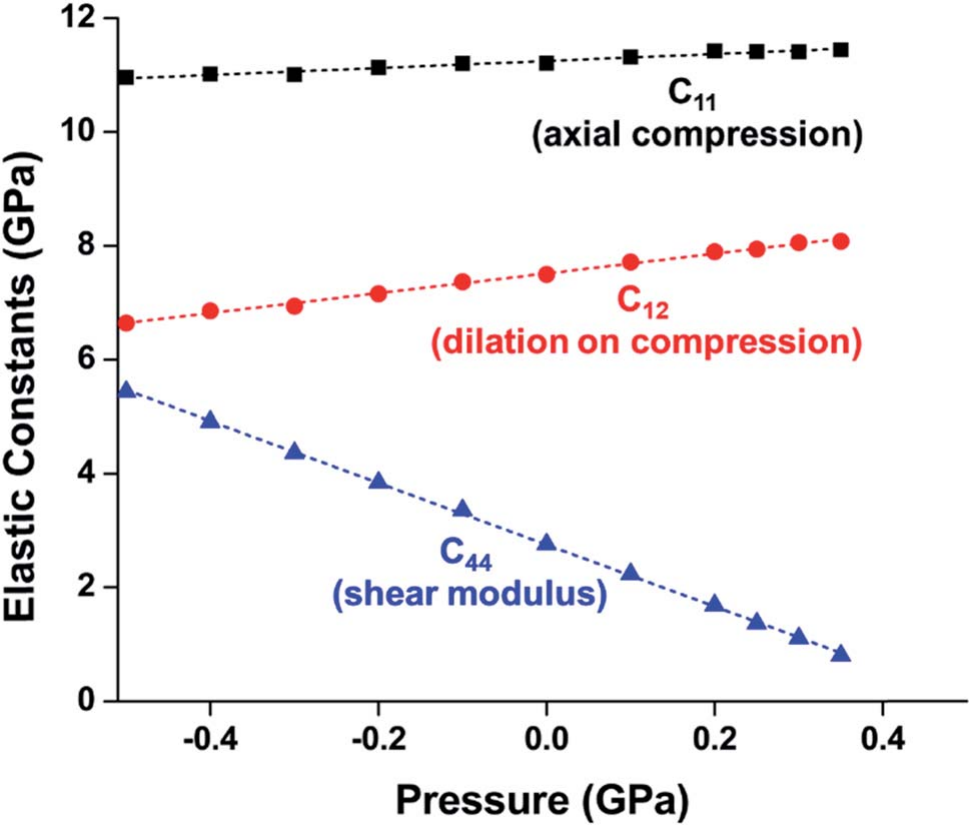
Plot of ZIF-8 unit cell parameter and elastic constants *vs.* pressure. *C*_11_, *C*_12_, and *C*_44_, correspond to the moduli for axial compression, dilation upon compression, and shear, respectively. The precipitous decline in *C*_44_ indicates a drop in the shear modulus at elevated pressure, a phenomenon known as “shear-mode softening”. Adapted with permission from ref. 24. Copyright 2013, American Chemical Society.

While experimental studies are essential for understanding the pressure response of MOFs, the insight that is gained from computational simulations is crucial for understanding the mechanism of compression and deformation. In 2013, Coudert and coworkers shed light on the mechanism of ZIF-8 and ZIF-4 amorphization through a molecular dynamics study.^24^ This investigation monitored the elastic constants that describe the mechanical properties of ZIF-8 as a function of pressure. Notably, the elastic constant corresponding to the shear modulus (*C*_44_) rapidly drops as the pressure is increased from 0.0 GPa to 0.35 GPa (Fig. 2), indicating that ZIF-8 becomes highly susceptible to shear forces as hydrostatic pressure increases. This behaviour is known as shear-mode softening and leads to the instability of the framework at pressures above 0.4 GPa. This study also indicates that ZIF-4 displays similar shear-mode softening, suggesting that this mechanism of amorphization may be generalizable to other ZIFs and MOFs.

**Fig. 3:**
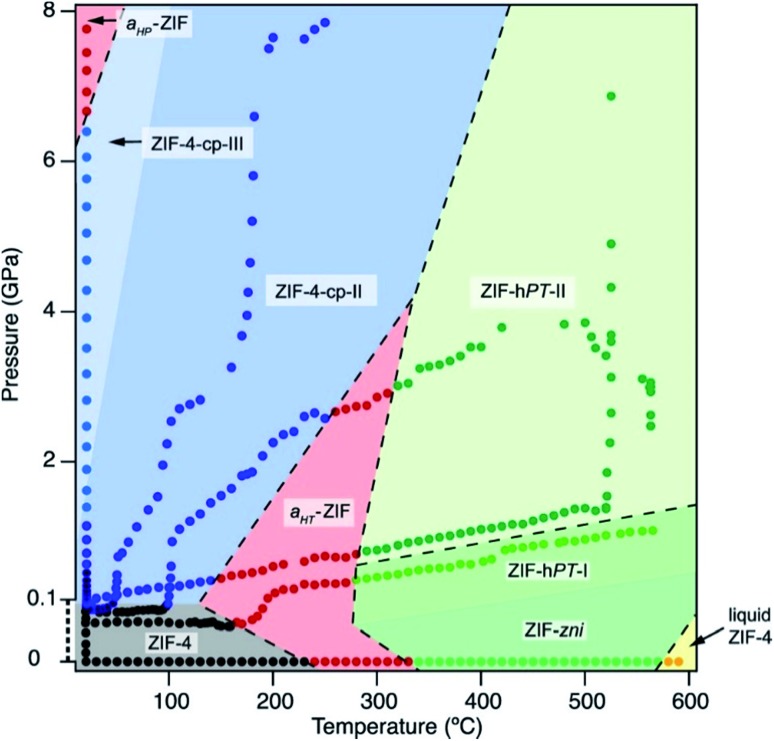
Pressure–temperature phase diagram of ZIF-4. The complexity of the diagram highlights the need for thorough studies to elucidate the numerous phases of MOFs. Reproduced with permission from ref. 28. Copyright 2019, American Chemical Society.

As the field of MOFs continues to advance, attention has been drawn to non-crystalline, glass^30^–^32^ and liquid^33^,^34^ phases of MOFs. In this area, Bennett and coworkers have explored the high-pressure and high-temperature phase diagram of ZIF-62 and ZIF-4 with both experimental and computational methods.^29^ Remarkably, this study reveals that the melting point of both ZIFs is lowered significantly at elevated hydrostatic pressures, opening the door to possible synthetic strategies to achieve liquid and glass MOFs that normally decompose before melting at ambient pressure. Further investigations into the high-pressure and high-temperature behaviour of ZIF-4 from Bennett and coworkers reveals four distinct high-pressure–high-temperature crystalline phases.^28^ The crystal structure of two of the phases were determined by PXRD refinement techniques, while structural refinement beyond space group and unit cell assignment was intractable for the other two phases. The phase diagram of ZIF-4 derived from this study is strikingly complex (Fig. 3), emphasizing the need for thorough studies to outline and understand the polymorphism of these frameworks. These exciting findings invite researchers to probe the high-pressure and high-temperature space for other classes of MOFs.

The examples highlighted here emphasize several pervasive characteristics of high-pressure studies of MOFs: (1) seemingly minor experimental details (*e.g.* choice of hydrostatic fluid) can have a drastic impact on results, (2) the combination of experiment and simulation is vital to build a deeper understanding of the processes at play at high pressure, and (3) the field of high-pressure–high-temperature behaviour of MOFs contains boundless space to explore in future efforts. As MOF technologies continue to advance and ZIFs become incorporated into composite materials, such as encapsulating enzymes and polymer fibres, the mechanical properties of these advanced materials will become a topic of interest. Moreover, the behaviour of ZIFs under dynamic pressures (*e.g.* shockwave or impact) has already gained attention^35^ and will continue to be an interesting area to examine for both fundamental understanding and practical applications.

## 3. Classic carboxylate MOFs

While the Cambridge Structural Database holds the structures of thousands of reported MOF structures,^36^ some of the “classic” frameworks have been the focus of a disproportionately large portion of research. Among these highly studied materials are HKUST-1 (also known as Cu-BTC), MOF-5 (also known as IRMOF-1), and the MIL series of MOFs. In this section, we will discuss each of these groups separately, though the insights gained from each material will hopefully help the community to shape the understanding of all MOFs.

### HKUST-1 (Cu-BTC)

In HKUST-1, dimeric Cu-paddlewheel metal nodes are bridged by 1,3,5-benzenetricarboxylate ligands to form a cubic framework that exhibits two types of pores. Among the first MOFs to be reported,^37^ HKUST-1 has become one of the prototypical carboxylate MOFs. As such, it has often been the subject of studies on the pressure response of these porous materials. Chapman *et al.* described two compression regimes observed by high-pressure PXRD,^38^ which were later confirmed and described in atomic detail by SCXRD analysis from Moggach and coworkers.^39^ Studies into the effect of pelletization by revealed that at pressures of 0.07 GPa can induce partial pore collapse in HKUST-1.^40^ While Peterson *et al.* attribute the significant hysteresis in the nitrogen sorption experiments of the pelletized sample to mesopore formation, the swift upward trend in the isotherm at *P*/*P*_0_ values near unity indicates the presence of large pores forming between the MOF crystallites, which may contribute to the observed hysteresis. Beyond high-pressure conditions, Heinen *et al.* demonstrated that, upon heating, both the hardness and Young's modulus of HKUST-1 drop steadily as the temperature rises from 25–100 °C.^41^ These findings are somewhat surprising in light of the negative thermal expansion of HKUST-1, meaning that although the MOF becomes more dense at high temperatures, the Young's modulus and hardness continue to decrease. In an interesting computational study, Dürholt, Keupp, and Schmid investigated the impact of missing-node defects in the crystal structure of HKUST-1 on the bulk modulus of the material. As expected, the presence of mesopores from these defects decreases the stability of the material; however, their calculations indicate that structures with a small number of larger mesopores exhibit a higher bulk modulus that those with many smaller mesopores exhibiting identical pore volume.^42^

**Fig. 4:**
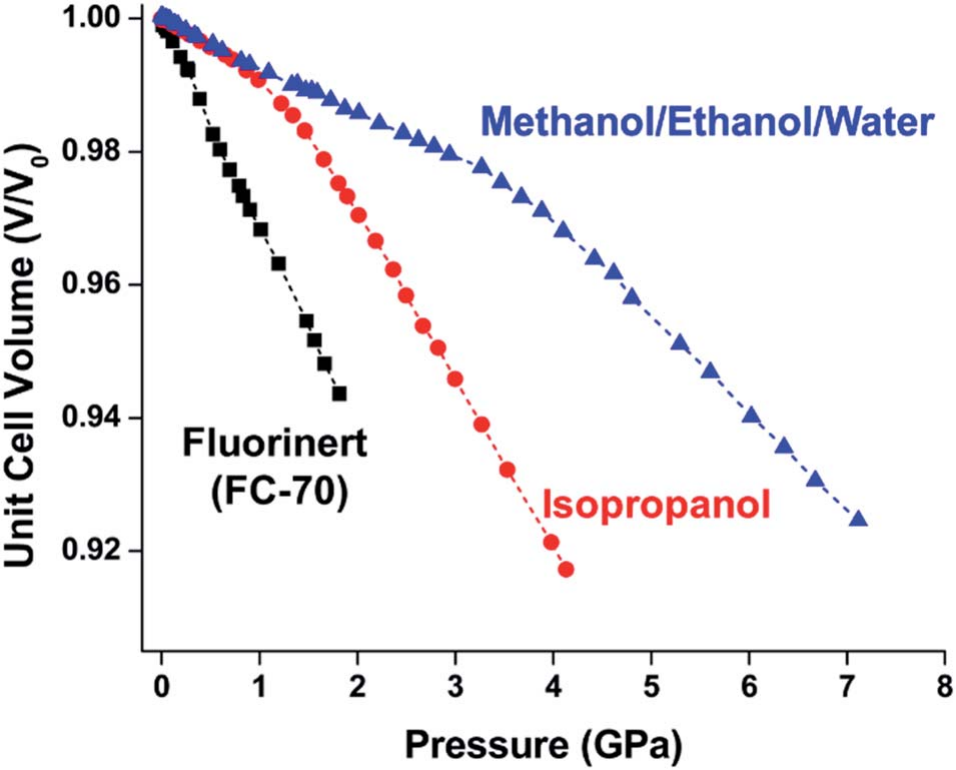
Changes in the unit cell volume of HKUST-1 as a function of pressure in the presence of different pressure transmitting fluids. The different initial slopes demonstrate the importance of non-penetrating fluids for the accurate determination of bulk moduli in MOFs. Adapted with permission from ref. 38. Copyright 2008, American Chemical Society.

In the first high-pressure diffraction study of HKUST-1, Chapman, Halder, and Chupas probed the compression of the MOF using PXRD in a DAC in the presence of different pressure-transmitting fluids.^38^ This important study first demonstrated the now well-known phenomenon of pressure-induced hyperfilling of MOF pores with sufficiently small fluids. For small-molecule fluids, such as isopropanol and methanol/ethanol/water mixtures, HKUST-1 exhibits two distinct regimes of compression: at lower pressures the apparent bulk modulus is exceptionally high (*K*_0_ = 117.6(1) GPa), while at higher pressure compression occurs at a more reasonably expected rate, with bulk moduli between 25.9(5) and 41.9(4) GPa. Conversely, when a non-penetrating pressure transmitting fluid (in this case, Fluorinert™ FC-70) is used, the observed bulk modulus is 29.5(7) GPa throughout the hydrostatic limit of the fluid. The plot of unit cell volume *vs.* pressure (Fig. 4) exemplifies the difference between pressure transmitting fluids. This work demonstrates the need for appropriate hydrostatic fluid selection to accurately determine the bulk modulus of the intrinsic framework.

### MOF-5 (IRMOF-1)

MOF-5 comprises tetranuclear Zn_4_(μ_4_O) nodes connected by 1,4-benzenedicarboxylate in an octahedral geometry, resulting in a simple cubic crystal structure. It was one of the first MOFs with high porosity,^43^ and due to the simplicity of the node, linker, and pore structure, MOF-5 has become the quintessential example of this class of porous materials. Moreover, this framework was the subject of one of the earliest studies concerning the mechanical properties of MOFs. Mattesini, Soler, and Ynduráin computed the bulk modulus and Young's modulus of MOF-5 in 2006, predicting that the material is readily compressible and soft, with a Young's modulus similar to that of Oak wood (14.8 GPa).^44^ Shortly thereafter, Allendorf and coworkers measured the Young's modulus using nanoindentation and found the experimental value to be even lower than their DFT calculations, which they attribute to deformation and buckling at the lowest loads applied in the experiment.^45^

**Fig. 5:**
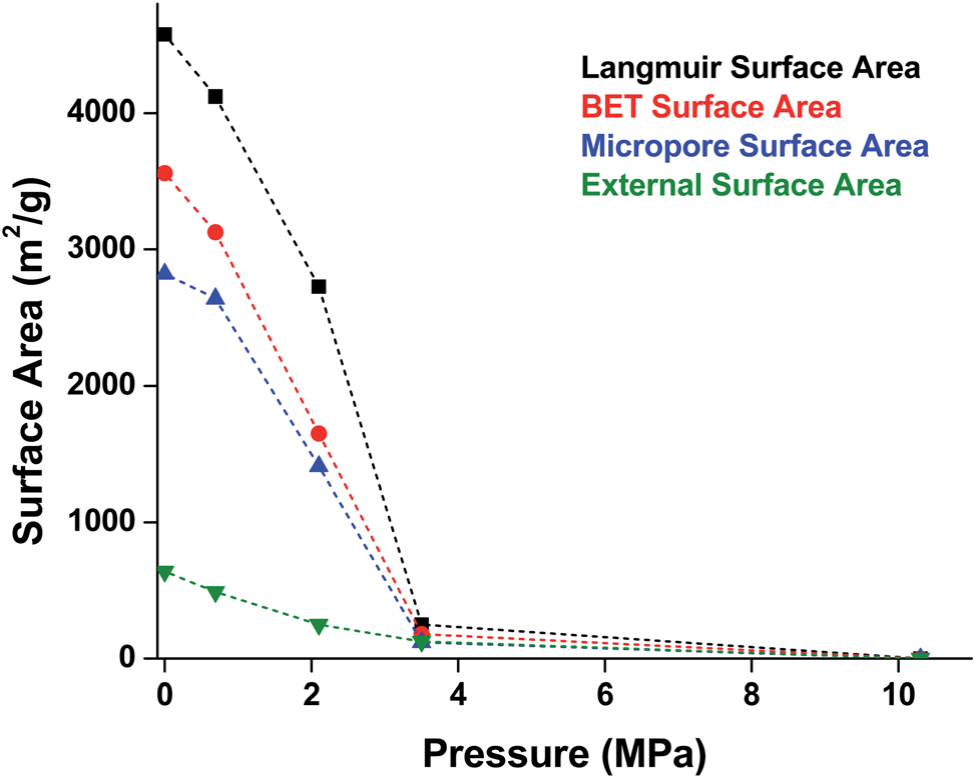
Surface area of MOF-5 as a function of pressure. Elevated pressures were obtained using an anvil press in the absence of pressure transmitting fluid. Surface area values were determined using N_2_ adsorption measurements at 77 K. Adapted with permission from ref. 47. Copyright 2010 by American Physical Society.

While Moggach and coworkers again observed pore hyperfilling in the presence of *N*,*N*-diethylformamide during SCXRD experiments in a DAC,^46^ Hu and Zhang found that MOF-5 undergoes rapid amorphization at pressures as low as 3.5 MPa in the absence of pressure-transmitting fluid.^47^ In this study, a number of *ex situ* experiments were conducted after exposure of MOF-5 to pressures ranging from 0.0 to 10.3 MPa. The loss of crystallinity observed by PXRD was corroborated by a steady decrease in the surface area as the pressure treatment increased (Fig. 5), indicating pressure-induced pore collapse. Furthermore, Raman spectroscopy revealed significant changes in the relative intensity of peaks after treatment with 10.3 MPa, further supporting structural changes under pressure. These results highlight the importance of pressure-transmitting fluid in studies of MOF compression, as the behaviour of these frameworks is highly dependent on the surrounding environment and the presence of fluid in the pores.

### MIL-53

Another class of well-studied carboxylate MOFs are the MILs (Matériaux de l’Institut Lavoisier). In particular, the wine-rack structure of MIL-53 has attracted a great deal of attention due to the ability to facilely vary the identity of the metal node and the flexibility of the MOF. Early experiments by Gascon and coworkers demonstrated the reversible compression of the flexible framework, NH_2_-MIL-53(In).^48^ Coudert and coworkers determined the mechanical properties of MIL-53(Al) and several other wine-rack type MILs through DFT calculations, demonstrating significant anisotropy in the Young's modulus that they attribute to the flexibility of the framework in two dimensions.^49^ Yot *et al.* conducted several studies of MILs with variations in the metal node identity and organic linker substitution, revealing phase transitions to different space groups^50^ and notable differences in the bulk moduli of the similar MOFs.^51^ Ramaswamy *et al*. investigated the compression of a fumaric acid analogue of MIL-53(Al) and MIL-53(Ga) at moderate pressures (up to 0.55 GPa) using computational and experimental methods.^52^ While they observed a phase transition in both frameworks at elevated pressure, this transition is found to be reversible in the Al analogue, but irreversible for the Ga MOF. DFT calculations corroborate this finding, revealing that the closed-pore phase of MIL-53(Ga) is a metastable state with an energy barrier five times greater than that of MIL-53(Al). This study emphasizes the potential for different behaviours between MOFs at high pressure, even for isostructural frameworks.

**Fig. 6:**
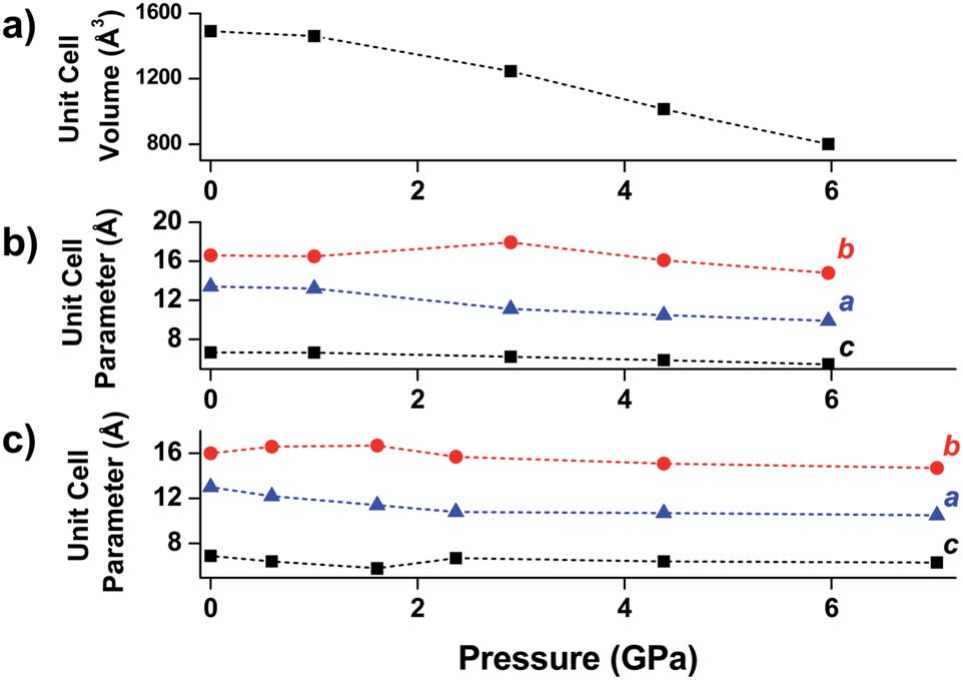
(a) Unit cell volume of MIL-53(Al), (b) unit cell parameters of MIL-53(Al), and (c) unit cell parameters of NH_2_-MIL-53(Al) as a function of pressure. The parameter corresponding to the *b*-axis exhibits a notable increase at low pressures—an example of negative linear compression. Adapted from ref. 54 – published by the Royal Society of Chemistry.

Wine-rack type MOFs have been predicted to exhibit interesting anisotropy in their elastic properties, and, in some cases, negative linear compressibility (*i.e.* expansion of a structure along one axis upon application of pressure) is expected to arise due to this anisotropy.^53^ This phenomenon was demonstrated experimentally by Serra-Crespo *et al.* in an X-ray diffraction study examining MIL-53(Al) and MIL-53-NH_2_(Al) in a DAC.^54^ While the unit cell volume decreases as expected upon increasing pressure, the individual unit cell parameters reveal interesting behaviour in both MOFs. Lattice parameters corresponding to the *a* and *c* crystallographic axes continuously decrease as a function of pressure, while the *b* axis, corresponding to the long axis of the diamond-shaped pores (Fig. 6), increases at low pressures (up to ∼3 GPa). This example of negative linear compressibility is an important step in understanding the mechanism of compression in MOFs and highlights the importance of exploring these phenomena further.

## 4. Zr-based MOFs

Among the greatest milestones in MOF synthesis and design of is the advent of hexanuclear Zr-based nodes. First reported in 2008,^55^ this Zr_6_ building block provides MOFs with exceptional chemical and thermal stability, enabling studies of MOFs in water, high temperatures, and other harsh conditions. Moreover, the high connectivity of the Zr_6_ node uncovers a plethora of diverse structures and topologies to be studied.^56^ As the first example of a Zr-based MOF, UiO-66 has become the prototypical framework for the entire class of materials and has been studied exhaustively. Here we summarize the efforts centred on UiO-66 as well as related MOFs (*e.g.* UiO-67) and others comprised of Zr nodes.

### UiO-66

The tetrahedral and octahedral cages of UiO-66 are the result of 12-connected Zr_6_ nodes joined by terephthalic acid (also known as 1,4-benzenedicarboxylic acid, or BDC). Early work measuring the shear modulus^57^ and effects of pelletization on the porosity^40^ of UiO-66 reveals a superior resistance to deformation and pore collapse than other carboxylate MOFs and ZIFs. The role of defect sites (*i.e.* locations in which a node or linker is missing from the ideal structure) was explored computationally by Coudert and coworkers, indicating that while defects increase the surface area and porosity of UiO-66, the absence of some structural components compromises the mechanical stability.^16^ Using atomic force microscopy techniques, Sun *et al.* demonstrated the importance of linker substitution on the elastic moduli of a series of UiO MOFs.^58^ Despite the higher bulk and shear moduli of UiO-66 relative to other MOFs, post-synthetic ball milling leads to rapid amorphization by breaking the coordination bonds that hold the framework together.^59^ In a spectroscopic study, Suslick and coworkers monitored the breakage of Zr–carboxylate bonds in UiO-66 at elevated pressures using infrared and X-ray absorption spectroscopies, finding that over half of these bonds were broken at 1.9 GPa.^60^ Suslick also conducted experiments to determine the energy storage of UiO-66 upon compression, revealing that MOFs may have potential applications as mechanical shock absorbers or dissipators.^61^

**Fig. 7:**
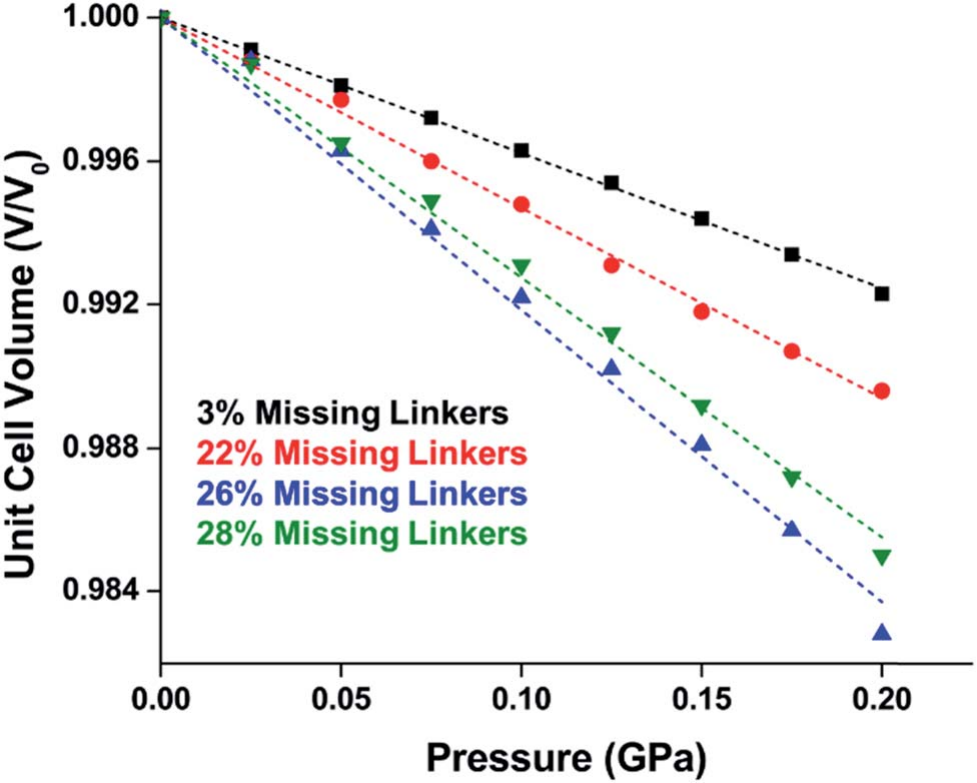
Unit cell volume of UiO-66 as a function of pressure. Each sample exhibits different defect density. A more shallow slope indicates a higher bulk modulus, while steeper slopes correspond to lower bulk modulus values. Adapted with permission from ref. 62. Copyright 2019, American Chemical Society.

Experimental evidence of the impact of defects on the compression of UiO-66 was reported by Dissegna *et al.*, corroborating Coudert's findings that increasing defect density generally yields a lower bulk modulus.^62^ By conducting these experiments in a water-filled cell rather than a traditional DAC, the pressure control at low pressures is exceptionally precise, allowing for measurements in 25 MPa intervals from 0–4 GPa. As the defect density increases from ∼3% to ∼26% of linkers missing, the bulk modulus decreases as expected; however, at higher defect densities (∼28% missing linkers), the bulk modulus of UiO-66 appears to increase (Fig. 7). This result comes as a surprise, inviting further experimental and computational study to understand how the presence of more extensive defects impacts the mechanical properties of MOFs.

### Other Zr MOFs

Beyond UiO-66, several analogues have been studied to probe the effects of changes in the linker length, flexibility, and steric bulk. In a joint computational and experimental venture, Bennett and coworkers demonstrated that the flexibility around the diazo group of UiO-abdc (abdc = 4,4′-azobenzene dicarboxylate) is responsible for the more rapid compression of the framework compared to the analogous UiO-67.^63^ Additionally, Marshall *et al.* examined the elastic moduli of UiO-type MOFs comprised of 4,4′-ethynylenedibenzoate (1) and 4,4′-stilbene dicarboxylate (2) linkers, along with their brominated analogues, revealing that increased linker flexibility decreases the elastic modulus of the materials.^64^ Efforts to explore the mechanical properties of more structurally diverse Zr-MOFs have been notably limited in the literature. Ryder, Civalleri, and Tan investigated the Young's modulus, shear modulus, linear compressibility, Poisson's ratio, and bulk modulus of an isoreticular series of MIL-140 [ZrO(BDC)] derivatives.^65^ This thorough computational study reveals that the Zr-MOFs offer numerous interesting structure–property relationships which can explain the observed amorphization of MIL-140 under ball-milling processing.^66^

**Fig. 8:**
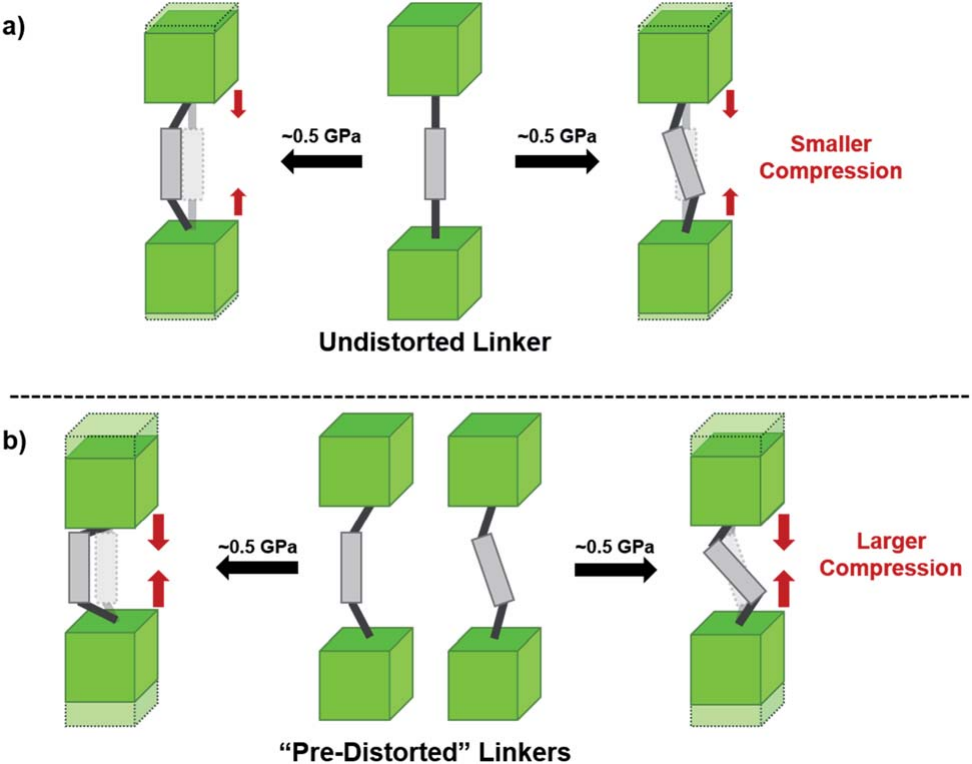
(a) MOFs with undistorted structures are more resistant to compression than (b) those with distortions in their structures at ambient pressure. Adapted with permission from ref. 67. Copyright 2019, American Chemical Society.

In the Farha group's first venture into the field of MOFs under pressure, we conducted a systematic study into the compression of two topological families of Zr-MOFs: UiO-type MOFs (fcu topology) and the NU-900 series of wine-rack type MOFs (scu topology).^67^ The aim of this study was to experimentally probe the effects of linker length and porosity across different topologies, allowing for more broadly generalizable conclusions to be drawn from the data. In general, we found that while linker length and nearest-node distance correlate well with bulk modulus for each series, only void fraction serves as a good predictor for both families of MOFs studied. Interestingly, two samples exhibited significantly lower bulk moduli than expected based on the compressibility of similar materials and previous computational results. Careful analysis of ambient condition single crystal structure models and unit cell volume changes reveal that the linkers of these two samples are “pre-distorted” and thereby do not resist compression as well as undistorted analogues (Fig. 8). It is worth noting that experimental surveys of the tremendous structural diversity of Zr-MOFs using DACs is currently hampered by the lack of appropriate non-penetrating pressure transmitting fluids for the numerous mesoporous frameworks. In order to properly determine the bulk modulus of these highly porous materials, the development of new pressure transmitting fluids is essential.

## 5. Other MOFs

With thousands of reported MOF structures,^68^ and more papers published every day, it would be impossible to categorize and measure the mechanical properties of every framework. Here we highlight several studies of MOFs that do not necessarily fit into the previous groupings but emphasize the breadth of interesting structures and phenomena reported.

While Zn(CN)_2_ normally forms a dense, non-porous structure, Lapidus *et al.* demonstrated that at elevated pressures (∼1 GPa) the crystal structure can undergo phase transitions to yield an open framework with lower density.^69^ Interestingly, these phase transitions depend greatly on the pressure transmitting fluid, emphasizing the role of the fluid in the mechanism of the transition. In a study of a 4,4′-bipyridine MOF with Co nodes (3), Zhou *et al.* observed a phase transition followed by negative linear compression.^70^ These studies are an example of the power of pressure to alter MOF structures and imbue new and desirable properties on the material.

Fundamental understanding of the mechanical properties of MOFs has led many to ask how these properties can be modified and controlled to suit the needs of different applications. Great effort has thus been put toward developing post-synthetic modifications that modulate the mechanical stability of MOFs. Henke, Li, and Cheetham demonstrate the anisotropy and guest-dependence of the elastic modulus and hardness of MOFs (4) pillared with DABCO (diazabicyclo[2.2.2]octane), indicating that simply modifying solvent choice can alter these properties.^71^ Several efforts have sought to improve the mechanical properties of MOF-74 by grafting the framework onto graphene or including *N*,*N*′-dimethylethylenediamine guest molecules in the pores.^72^,^73^ While the gas sorption properties remained fairly similar, these post-synthetic processes have an impact on the elastic modulus, hardness, Young's modulus, and shear modulus.

In the ever-expanding field of porous materials, new and fascinating materials are continuously created and characterized. Recently, Lal *et al.* synthetized an interesting MOF-like material (5) by cross-linking the dangling ligands on metal–organic polyhedra.^74^ While this structure is far from a traditional, crystalline MOF, nanoindentation studies reveal that its hardness correlates with the crosslinking density, akin to trends seen in other MOFs with additional supporting linkers between metal nodes. As new materials are developed, the foundational knowledge gained through years of studying mechanical properties of MOFs and other porous materials will help to guide and direct the design of these structures.

## 6. Future directions

Since the discovery of the first highly porous MOFs twenty years ago, the field of synthesis, characterization, and design of these scaffolds has been nothing short of prodigious. Rational synthesis of targeted materials designed with specific applications in mind has proven to be an effective strategy for developing new materials with desirable structural and chemical properties. As studies of the mechanical properties of MOFs continue, we hope that similar “design rules” can be developed for these characteristics as well. While trends, such as the inverse relationship between porosity and bulk modulus, have been demonstrated previously, more nuanced factors like linker dimensionality, node binding motifs, and distortions in cluster-based nodes require more careful study to begin to tease out reliable structure–property relationships. As more detailed design rules are established, further examination of the interplay between these structural trends will enable a wholistic, predictive model of the mechanical properties of new materials. Hönicke *et al.* exemplified the importance of such a predictive model in a recent article. In this work, the bulk and shear moduli of a new ultrahigh porosity MOF (DUT-60) were estimated computationally prior to synthesis to ensure that it could survive activation.^75^ We anticipate that the behaviour of MOFs under mechanical stress will become a key parameter for the design and optimization of these materials in both fundamental and industrial settings.

**Fig. 9:**
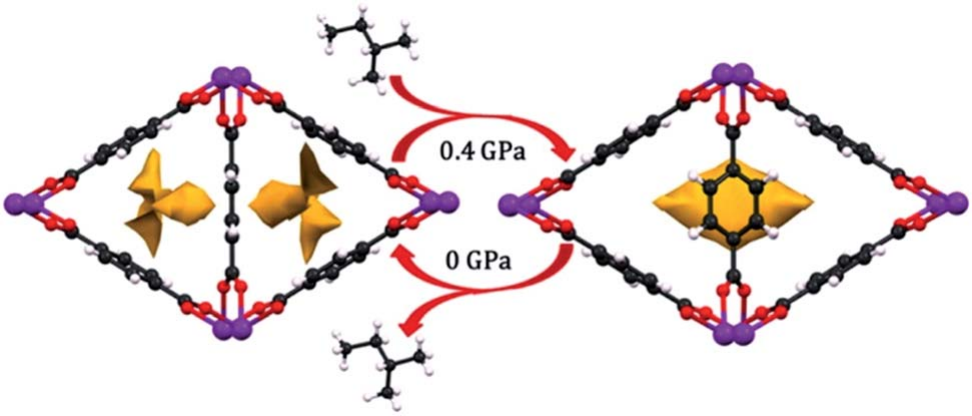
Pore shape modification of Sc_2_BDC_3_ upon exposure to 2-methylbutane at elevated pressure. Rotation of the phenyl rings reveals a larger cavity that can accommodate molecules that cannot fit in the pores at ambient conditions. Reprinted with permission from ref. 77. Copyright 2015, American Chemical Society.

Among the most interesting phenomena observed in the articles discussed above are pressure-induced phase transitions. While ball milling has been a long-established method for MOF synthesis,^76^ the application of pressure to achieve new phases of these scaffolds presents largely unexplored synthetic opportunities. Recently, Moggach and coworkers observed structural changes to a Sc-based MOF (Sc_2_BDC_3_) that alters its pore shape to accommodate molecules (*e.g.* 2-methylbutane) that cannot fit inside the MOF under ambient conditions (Fig. 9).^77^ This, along with the introduction of porosity into Zn(CN),^69^ are shining examples of the power of pressure to achieve new structures with desirable properties, rather than acting only as a destructive force that results in amorphization. Given the propensity for pore hyperfilling in MOFs at elevated pressure, we envision the potential for controlled phase transitions from MOFs comprised of 2-D sheets to 3-D structures and *vice versa*. Understanding the role of pressure in the formation of new MOF phases will enable the synthesis of novel materials that cannot be obtained with traditional solvothermal synthesis.

**Fig. 10:**
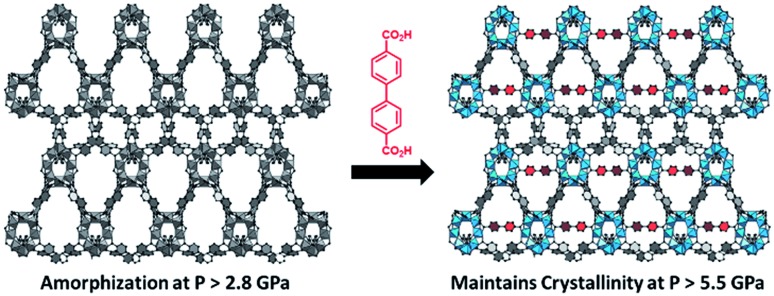
MOF-520 (left) demonstrates improved stability under pressure after incorporation of 4,4′-biphenyldicarboxylic acid “girder” linkers (right). Adapted with permission from ref. 80. Copyright 2017, American Chemical Society.

While the flexibility of the organic linker in MOFs has been shown to correlate with compression of the material, recent synthetic efforts have revealed numerous examples of frameworks with inherent flexibility under mild stimuli, such as changing solvent polarity.^78^,^79^ Investigating the mechanical properties of these highly flexible frameworks is crucial to understand the differences between these pliable scaffolds and more rigid MOFs. We envision two strategies for designing materials that can withstand high pressures: reinforce the structure to resist compression, or intentionally include a “bend, don't break” motif that allows the MOF to deform under pressure and rebound to the original structure upon release of the pressure. In an example of structural reinforcement, Yaghi and coworkers recently demonstrated the viability of “molecular retrofitting” to improve the stability of a framework that readily undergoes amorphization under pressure.^80^ By installing 4,4′-biphenyldicarboxylate into MOF-520 [Al_8_(μ-OH)_8_(HCOO)_4_(1,3,5-benzenetribenzoate)_4_], crystallinity was maintained up to 5.5 GPa, while the pristine MOF amorphized at pressures over 2.8 GPa (Fig. 10). On the other hand, the capacity for flexibility to impart structural resilience and maintain MOF functionality requires further study.

## 7. Conclusions

As the design and application of MOFs become more sophisticated, the importance of a fundamental understanding of mechanical properties in these porous frameworks grows as well. Furthermore, MOFs are at a turning point for their transition to industrial relevance,^81^ with post-synthetic processability standing out as a point of possible concern. For example, the formation of pellets while maintaining structural integrity is crucial to facilitate safe handling and to limit backpressure in packed columns for gas sorption. We envision that fundamental advances in this area will lead to design-principles that enable the commercialization of new MOF technologies. Here, we have outlined several studies that contributed to the current understanding of MOF behaviour under mechanical stress. Additionally, we suggest some interesting areas for future studies to continue to expand the understanding of the community and overcome current limitations in experimental design. Despite the tremendous work in this area, the ever-growing pool of new materials and the rich structural diversity of MOFs will continue to supply interesting and important specimens to study.

## Conflicts of interest

There are no conflicts to declare.

## References

[cit1] Li B., Wen H.-M., Zhou W., Chen B. (2014). J. Phys. Chem. Lett..

[cit2] Lee J., Farha O. K., Roberts J., Scheidt K. A., Nguyen S. T., Hupp J. T. (2009). Chem. Soc. Rev..

[cit3] Qiu S., Xue M., Zhu G. (2014). Chem. Soc. Rev..

[cit4] Wu M.-X., Yang Y.-W. (2017). Adv. Mater..

[cit5] Hindocha S., Poulston S. (2017). Faraday Discuss..

[cit6] Hazen R. M. (1983). Science.

[cit7] Wu J., Abu-Omar M. M., Tolbert S. H. (2001). Nano Lett..

[cit8] Lee Y., Vogt T., Hriljac J. A., Parise J. B., Hanson J. C., Kim S. J. (2002). Nature.

[cit9] Colligan M., Forster P. M., Cheetham A. K., Lee Y., Vogt T., Hriljac J. A. (2004). J. Am. Chem. Soc..

[cit10] Hriljac J. A. (2006). Crystallogr. Rev..

[cit11] Coudert F.-X., Fuchs A. H. (2016). Coord. Chem. Rev..

[cit12] Moghadam P. Z., Rogge S. M. J., Li A., Chow C.-M., Wieme J., Moharrami N., Aragones-Anglada M., Conduit G., Gomez-Gualdron D. A., Van Speybroeck V., Fairen-Jimenez D. (2019). Matter.

[cit13] Tan J. C., Cheetham A. K. (2011). Chem. Soc. Rev..

[cit14] McKellar S. C., Moggach S. A. (2015). Acta Crystallogr., Sect. B: Struct. Sci., Cryst. Eng. Mater..

[cit15] Burtch N. C., Heinen J., Bennett T. D., Dubbeldam D., Allendorf M. D. (2018). Adv. Mater..

[cit16] Bennett T. D., Cheetham A. K., Fuchs A. H., Coudert F. X. (2016). Nat. Chem..

[cit17] Katrusiak A. (2008). Acta Crystallogr., Sect. A: Found. Crystallogr..

[cit18] Pan Y., Liu Y., Zeng G., Zhao L., Lai Z. (2011). Chem. Commun..

[cit19] Yuan S., Feng L., Wang K., Pang J., Bosch M., Lollar C., Sun Y., Qin J., Yang X., Zhang P., Wang Q., Zou L., Zhang Y., Zhang L., Fang Y., Li J., Zhou H.-C. (2018). Adv. Mater..

[cit20] Moggach S. A., Bennett T. D., Cheetham A. K. (2009). Angew. Chem., Int. Ed..

[cit21] Chapman K. W., Halder G. J., Chupas P. J. (2009). J. Am. Chem. Soc..

[cit22] Tan J. C., Bennett T. D., Cheetham A. K. (2010). Proc. Natl. Acad. Sci. U. S. A..

[cit23] Bennett T. D., Simoncic P., Moggach S. A., Gozzo F., Macchi P., Keen D. A., Tan J.-C., Cheetham A. K. (2011). Chem. Commun..

[cit24] Ortiz A. U., Boutin A., Fuchs A. H., Coudert F.-X. (2013). J. Phys. Chem. Lett..

[cit25] Ryder M. R., Tan J.-C. (2016). Dalton Trans..

[cit26] Gao H., Wei W., Dong L., Feng G., Jiang X., Wu R., Lin Z., Li W. (2017). Crystals.

[cit27] Moosavi S. M., Boyd P. G., Sarkisov L., Smit B. (2018). ACS Cent. Sci..

[cit28] Widmer R. N., Lampronti G. I., Chibani S., Wilson C. W., Anzellini S., Farsang S., Kleppe A. K., Casati N. P. M., MacLeod S. G., Redfern S. A. T., Coudert F. X., Bennett T. D. (2019). J. Am. Chem. Soc..

[cit29] Widmer R. N., Lampronti G. I., Anzellini S., Gaillac R., Farsang S., Zhou C., Belenguer A. M., Wilson C. W., Palmer H., Kleppe A. K., Wharmby M. T., Yu X., Cohen S. M., Telfer S. G., Redfern S. A. T., Coudert F.-X., MacLeod S. G., Bennett T. D. (2019). Nat. Mater..

[cit30] Zhao Y., Lee S.-Y., Becknell N., Yaghi O. M., Angell C. A. (2016). J. Am. Chem. Soc..

[cit31] Bennett T. D., Yue Y., Li P., Qiao A., Tao H., Greaves N. G., Richards T., Lampronti G. I., Redfern S. A. T., Blanc F., Farha O. K., Hupp J. T., Cheetham A. K., Keen D. A. (2016). J. Am. Chem. Soc..

[cit32] Bennett T. D., Tan J.-C., Yue Y., Baxter E., Ducati C., Terrill N. J., Yeung H. H. M., Zhou Z., Chen W., Henke S., Cheetham A. K., Greaves G. N. (2015). Nat. Commun..

[cit33] Gaillac R., Pullumbi P., Beyer K. A., Chapman K. W., Keen D. A., Bennett T. D., Coudert F.-X. (2017). Nat. Mater..

[cit34] Umeyama D., Horike S., Inukai M., Itakura T., Kitagawa S. (2015). J. Am. Chem. Soc..

[cit35] Zhou X., Miao Y.-R., Shaw W. L., Suslick K. S., Dlott D. D. (2019). J. Am. Chem. Soc..

[cit36] Moghadam P. Z., Li A., Wiggin S. B., Tao A., Maloney A. G. P., Wood P. A., Ward S. C., Fairen-Jimenez D. (2017). Chem. Mater..

[cit37] Chui S. S. Y., Lo S. M. F., Charmant J. P. H., Orpen A. G., Williams I. D. (1999). Science.

[cit38] Chapman K. W., Halder G. J., Chupas P. J. (2008). J. Am. Chem. Soc..

[cit39] Graham A. J., Tan J.-C., Allan D. R., Moggach S. A. (2012). Chem. Commun..

[cit40] Peterson G. W., DeCoste J. B., Glover T. G., Huang Y., Jasuja H., Walton K. S. (2013). Microporous Mesoporous Mater..

[cit41] Heinen J., Ready A. D., Bennett T. D., Dubbeldam D., Friddle R. W., Burtch N. C. (2018). ACS Appl. Mater. Interfaces.

[cit42] Dürholt J. P., Keupp J., Schmid R. (2016). Eur. J. Inorg. Chem..

[cit43] Li H., Eddaoudi M., O'Keeffe M., Yaghi O. M. (1999). Nature.

[cit44] Mattesini M., Soler J. M., Ynduráin F. (2006). Phys. Rev. B: Condens. Matter Mater. Phys..

[cit45] Bahr D. F., Reid J. A., Mook W. M., Bauer C. A., Stumpf R., Skulan A. J., Moody N. R., Simmons B. A., Shindel M. M., Allendorf M. D. (2007). Phys. Rev. B: Condens. Matter Mater. Phys..

[cit46] Graham A. J., Allan D. R., Muszkiewicz A., Morrison C. A., Moggach S. A. (2011). Angew. Chem., Int. Ed..

[cit47] Hu Y. H., Zhang L. (2010). Phys. Rev. B: Condens. Matter Mater. Phys..

[cit48] Serra-Crespo P., Stavitski E., Kapteijn F., Gascon J. (2012). RSC Adv..

[cit49] Ortiz A. U., Boutin A., Fuchs A. H., Coudert F.-X. (2013). J. Chem. Phys..

[cit50] Yot P. G., Yang K., Guillerm V., Ragon F., Dmitriev V., Parisiades P., Elkaïm E., Devic T., Horcajada P., Serre C., Stock N., Mowat J. P. S., Wright P. A., Férey G., Maurin G. (2016). Eur. J. Inorg. Chem..

[cit51] Yot P. G., Yang K., Ragon F., Dmitriev V., Devic T., Horcajada P., Serre C., Maurin G. (2016). Dalton Trans..

[cit52] Ramaswamy P., Wieme J., Alvarez E., Vanduyfhuys L., Itié J.-P., Fabry P., Van Speybroeck V., Serre C., Yot P. G., Maurin G. (2017). J. Mater. Chem. A.

[cit53] Ortiz A. U., Boutin A., Fuchs A. H., Coudert F.-X. (2012). Phys. Rev. Lett..

[cit54] Serra-Crespo P., Dikhtiarenko A., Stavitski E., Juan-Alcañiz J., Kapteijn F., Coudert F.-X., Gascon J. (2015). CrystEngComm.

[cit55] Cavka J. H., Jakobsen S., Olsbye U., Guillou N., Lamberti C., Bordiga S., Lillerud K. P. (2008). J. Am. Chem. Soc..

[cit56] Chen Z., Hanna S. L., Redfern L. R., Alezi D., Islamoglu T., Farha O. K. (2019). Coord. Chem. Rev..

[cit57] Wu H., Yildirim T., Zhou W. (2013). J. Phys. Chem. Lett..

[cit58] Sun Y., Hu Z., Zhao D., Zeng K. (2017). ACS Appl. Mater. Interfaces.

[cit59] Bennett T. D., Todorova T. K., Baxter E. F., Reid D. G., Gervais C., Bueken B., Van de Voorde B., De Vos D., Keen D. A., Mellot-Draznieks C. (2016). Phys. Chem. Chem. Phys..

[cit60] Su Z., Miao Y.-R., Zhang G., Miller J. T., Suslick K. S. (2017). Chem. Sci..

[cit61] Miao Y.-R., Su Z., Suslick K. S. (2017). J. Am. Chem. Soc..

[cit62] Dissegna S., Vervoorts P., Hobday C. L., Düren T., Daisenberger D., Smith A. J., Fischer R. A., Kieslich G. (2018). J. Am. Chem. Soc..

[cit63] Hobday Claire L., Marshall Ross J., Murphie Colin F., Sotelo J., Richards T., Allan David R., Düren T., Coudert F.-X., Forgan Ross S., Morrison Carole A., Moggach Stephen A., Bennett Thomas D. (2016). Angew. Chem., Int. Ed..

[cit64] Marshall R. J., Richards T., Hobday C. L., Murphie C. F., Wilson C., Moggach S. A., Bennett T. D., Forgan R. S. (2016). Dalton Trans..

[cit65] Ryder M. R., Civalleri B., Tan J.-C. (2016). Phys. Chem. Chem. Phys..

[cit66] Van de Voorde B., Stassen I., Bueken B., Vermoortele F., De Vos D., Ameloot R., Tan J.-C., Bennett T. D. (2015). J. Mater. Chem. A.

[cit67] Redfern L. R., Robison L., Wasson M. C., Goswami S., Lyu J., Islamoglu T., Chapman K. W., Farha O. K. (2019). J. Am. Chem. Soc..

[cit68] Nazarian D., Camp J. S., Chung Y. G., Snurr R. Q., Sholl D. S. (2017). Chem. Mater..

[cit69] Lapidus S. H., Halder G. J., Chupas P. J., Chapman K. W. (2013). J. Am. Chem. Soc..

[cit70] Zhou M., Wang K., Men Z., Sun C., Li Z., Liu B., Zou G., Zou B. (2014). CrystEngComm.

[cit71] Henke S., Li W., Cheetham A. K. (2014). Chem. Sci..

[cit72] Kumar R., Raut D., Ramamurty U., Rao C. N. R. (2016). Angew. Chem., Int. Ed..

[cit73] Lee J.-H., Siegelman R. L., Maserati L., Rangel T., Helms B. A., Long J. R., Neaton J. B. (2018). Chem. Sci..

[cit74] Lal G., Derakhshandeh M., Akhtar F., Spasyuk D. M., Lin J.-B., Trifkovic M., Shimizu G. K. H. (2019). J. Am. Chem. Soc..

[cit75] Hönicke I. M., Senkovska I., Bon V., Baburin I. A., Bönisch N., Raschke S., Evans J. D., Kaskel S. (2018). Angew. Chem., Int. Ed..

[cit76] Pichon A., Lazuen-Garay A., James S. L. (2006). CrystEngComm.

[cit77] McKellar S. C., Sotelo J., Greenaway A., Mowat J. P. S., Kvam O., Morrison C. A., Wright P. A., Moggach S. A. (2016). Chem. Mater..

[cit78] Zhang Y., Zhang X., Lyu J., Otake K.-i., Wang X., Redfern L. R., Malliakas C. D., Li Z., Islamoglu T., Wang B., Farha O. K. (2018). J. Am. Chem. Soc..

[cit79] Hanna S. L., Zhang X., Otake K.-i., Drout R. J., Li P., Islamoglu T., Farha O. K. (2019). Cryst. Growth Des..

[cit80] Kapustin E. A., Lee S., Alshammari A. S., Yaghi O. M. (2017). ACS Cent. Sci..

[cit81] Faust T. (2016). Nat. Chem..

